# Effects of Anthocyanins in Composite Meals on Cardiometabolic Outcomes—A Systematic Review of Randomized Controlled Feeding Trials

**DOI:** 10.3390/nu12123781

**Published:** 2020-12-09

**Authors:** Jun Leong Sean Ou, Dimeng Yang, Mei Hui Liu

**Affiliations:** 1 Division of Endocrinology, Department of Medicine, Yong Loo Lin School of Medicine, National University of Singapore, 14 Medical Drive, Singapore 117599, Singapore; mdcojls@nus.edu.sg (J.L.S.O.); mdcyd@nus.edu.sg (D.Y.); 2 Department of Food Science and Technology, National University of Singapore, Science Drive 2, Singapore 117543, Singapore

**Keywords:** anthocyanin, composite meal, cardiovascular, metabolic, human nutritional intervention

## Abstract

Accumulating epidemiological evidence suggests that anthocyanin intake is associated with reduced risks of cardiometabolic disorders, highlighting the importance of incorporating the phytochemical in our diets. Numerous food-based intervention studies have examined, in controlled meal settings, the role of anthocyanin on cardiometabolic health; but their effects have not been systematically summarized. This study aims to systematically review and summarize the effects of anthocyanin consumption with composite meals on cardiometabolic health from randomized controlled feeding trials. A systematic literature search for relevant human nutritional intervention studies was performed using PubMed, Embase, Cochrane Library, CINAHL Plus with Full Text, and Scopus databases. The Cochrane Risk of Bias tool was used to assess the study quality. Eighteen articles involving 371 participants were included in this review. Consistent improvements from anthocyanin intake were found in glycemic, gastric inhibitory peptide (GIP), interleukin-6 (IL-6), and oxygen radical absorbance capacity (ORAC) responses. Anthocyanin intake did not significantly affect other markers of energy metabolism, vascular functions, oxidative stress and antioxidant status, as well as inflammatory responses. Inconsistencies in successful outcomes between epidemiological studies and included interventions were largely attributed to matrix effects, which may impede the bioaccessibility of anthocyanins and consequently, limiting its health benefits when co-delivered with some foods.

## 1. Introduction

Metabolic syndrome is a cluster of physiological disorders that play a major role in pathological complications of type 2 diabetes (T2D) and cardiovascular diseases (CVD). Dietary modifications and supplementations have been recognized as cost-effective preventive and management strategies against metabolic syndrome [1]. Anthocyanins are a class of water-soluble flavonoids that have been well represented in the human diet and have garnered interest due to their health-promoting properties. These phytochemicals are responsible for the red, blue and violet hues produced naturally; and are widely distributed in fruits, vegetables, flowers, and some grains [2].

Epidemiological evidence has established that increasing dietary anthocyanin intake from the consumption of whole or processed foods, and supplementation, may lower the risk of cardiometabolic diseases [3,4,5,6]. Several reviews have also summarized the beneficial effects of dietary anthocyanins on insulin resistance [7], dyslipidemia [8], vascular health [9], oxidative stress [7] and inflammation [10]. Evidence from these studies, with administered doses between 1.65 to 1323 mg/day, support the potential of anthocyanins to mitigate the onset of metabolic diseases in a dose-dependent manner. 

While some intervention studies confirm the reported health benefits, others had findings which were inconsistent with epidemiological evidence. An 8-week blueberry supplementation was found to have no significant impact on glycemic and lipidemic responses [11]. Another study reported no improvements in vascular functions and oxidative stress over 2 h after the consumption of a blackcurrant extract [12]. It was suggested that these inconsistencies may be attributed to dietary factors that confound the outcomes, such as the presence of other macronutrients in dietary interventions [13].

Therefore, this review aims to assess the effects of anthocyanin consumption on outcomes of cardiometabolic health in composite meal settings. Additionally, the discrepancies between outcomes of anthocyanin consumption in epidemiological studies versus intervention trials will be evaluated. 

## 2. Materials and Methods

The current systematic review was performed according to the Preferred Reporting Items for Systematic Review and Meta-Analysis guidelines [14]. The protocol was registered in the PROSPERO international prospective register of systematic review (CRD42020157432). 

### 2.1. Search Strategy

A systematic literature search was performed in the PubMed, EMBASE, Cochrane Library, CINAHL Plus with Full Text, and Scopus databases, identifying relevant English-language articles up to 21 November 2019. Potentially eligible articles were independently screened by 2 reviewers (SJLO and DY) based on their titles, abstracts, and full texts. Duplicate references found in the initial search were excluded. Original articles fulfilling the following eligibility criteria were then retrieved: (1) populations including adults of at least 18 years; (2) interventions including composite meal challenges (meal with several food items) with purified anthocyanins or anthocyanin-rich foods; (3) use of a relevant control or placebo; (4) outcomes including cardiometabolic markers; (5) randomized controlled nutritional interventions of full-feeding designs (study team provides all food materials). See Table S1 for the full electronic search strategy. In addition to not fulfilling the eligibility criteria, articles were excluded if anthocyanins were not the primary bioactive detected, or if they did not report the dose of anthocyanins used. Additionally, each reviewer conducted a manual search of reference lists from the retrieved original articles and recent reviews. Contrasting opinions between the 2 reviewers regarding eligibility were resolved by consensus.

### 2.2. Data Extraction

Data from the included articles were extracted and double checked by the 2 reviewers. Differences were resolved by discussion with a third reviewer (MHL). The following information was extracted from included articles: (1) first author, year; (2) country of origin; (3) study design; (4) population size; (5) mean age; (6) health status; (7) intervention length; (8) meal composition; (9) anthocyanin source; (10) anthocyanin dose or equivalent dose; (11) control; (12) study outcomes; and (13) study results.

### 2.3. Quality Assessment Criteria

The quality of included articles was assessed according to the criteria of Cochrane Handbook for Systematic Reviews of Interventions [15]. The following methodological domains were considered: (1) random sequence generation; (2) allocation concealment; (3) blinding of participants and personnel; (4) blinding of outcome assessment; (5) incomplete outcome data; and (6) selective reporting. Each domain was evaluated and classified as having a low, high, or unclear risk of bias.

## 3. Results

### 3.1. Literature Search and Study Selection

**Figure 1:**
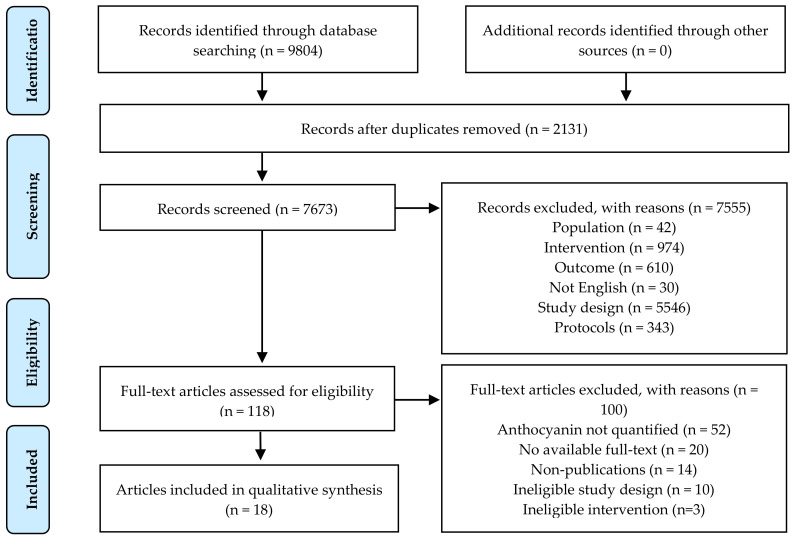
PRISMA flow diagram for the literature search and article selection.

The PRISMA Flow Diagram for the literature search and article selection process is presented in Figure 1. Briefly, a total of 9804 potential articles were identified after the initial search, of which 2131 duplicates were excluded. Of the remaining 7673 articles, title and abstract screening excluded 7555 articles for not meeting the inclusion criteria. Even though our search strategy had attempted to only include human studies, 5546 articles were still found to be of inappropriate study designs among the excluded studies (n = 7555), such as reviews and observational studies. Additionally, our review aimed to only include studies in which anthocyanins were given with composite test meals. Hence, we also excluded 974 articles which used interventions that fell outside our scope. The remaining 118 articles were reviewed in full text and 100 were excluded for: anthocyanins not primary or quantified (n = 53), no available full-text (n = 20), non-publications (n = 14), ineligible study design (n = 10), and ineligible intervention (n = 3). No additional articles were identified from hand searching reference lists. Thus, 18 eligible articles were included in the systematic review.

### 3.2. Study Characteristics

The characteristics of included articles are summarized in Table 1. Overall, 18 articles were included in the systematic review, of which 8 were conducted in the United States of America [16,17,18,19,20,21,22,23], 3 in Italy [24,25,26], 3 in the United Kingdom [12,27,28], and 2 in Chile [29,30]. One study each was conducted in Canada [31], and Austria [32].

#### 3.2.1. Population Characteristics

A total of 371 participants were represented in the 18 included articles, with numbers between 6 and 34 participants. Of the 18 eligible articles, 20 studies were described: 2 studies had exclusively male participants [28,29], 1 included women only [22], 12 included both males and females [12,16,17,18,19,20,23,26,27,31,32], and 4 did not report genders [21,24,25,32]. Most studies (n = 10) recruited middle-aged adults (mean age 36–55) [12,17,18,19,20,22,25,26,28,31], 8 studies involved young adults (mean age 18–35) [16,20,21,23,24,27,29,32]. Mean age was not reported in 1 study [30]. The health statuses of participants varied—9 studies recruited healthy participants [12,21,22,23,24,27,29,31,32] and 3 were restricted to the overweight or obese [19,25,26]. One study each included the healthy, obese, and overweight [16]; the obese with insulin resistance [17]; the obese and diabetic [18], and those with 1–10% CVD risk in 10 years, as determined by QRISK 2 [28]. Three studies did not report the health status of their participants [20,30].

#### 3.2.2. Study Design

Of the 20 included randomized controlled trials (RCT), 18 studies were crossover [12,16,17,18,19,20,21,22,24,25,26,27,28,29,30,31,32] and 2 were parallel designs [23,32]. Nineteen studies were acute [12,16,17,18,19,20,21,22,23,24,25,26,27,28,29,30,31,32], with study durations ranging from 2 to 24 h. One article reported both chronic (2-week) and acute (6-h) nutritional interventions on separate occasions [32]. 

#### 3.2.3. Intervention Characteristics

Macronutrient compositions of meal challenges and full-feeding diets in the 20 eligible studies mostly varied the carbohydrate and fat contents. Meals and diets in 11 studies were representative of a high-carbohydrate (HC, >40% of calories from carbohydrates) meal [12,17,19,20,21,22,27,30,31,32], 7 were high-fat (HF, >40% of calories from fats) [16,18,24,25,26,28,29], and 1 was high-carbohydrate and high-fat (HC/HF, >40% of calories each from carbohydrates and fats) [23]. There were no studies with high-protein (HP, >40% of calories each from proteins) meals or diets.

Sources of anthocyanin used in eligible studies were mostly fruit concentrates (n = 12) [12,16,17,19,21,22,23,27,29,30,32]. Other sources included 4 studies that used whole fruits [18,20,31], 2 that used fruit-based beverages [25,26], 1 that used red wine [24], and 1 that used a flower-based beverage [28]. Total anthocyanin intake ranged from 32 mg/day to 600 mg/day—15 studies provided a maximum dose of ≤300 mg/day [16,17,18,19,20,21,22,23,25,26,28,29,30,32], and 4 studies with > 300 mg/day [12,27,31,32]. The absolute dose of anthocyanins was not determinable in 1 study [24]. All studies compared the intervention to a placebo or control.

#### 3.2.4. Control Groups

Of the 20 studies, 16 were placebo-controlled. These included 11 studies with calorie-matched placebo beverages and meals [12,16,17,18,19,21,23,25,26,27,30], 3 with placebo gels and gel capsules [31,32], and 2 with water [28,29]. Four studies were controlled, but without the provision of placebos [20,22,24].

#### 3.2.5. Outcome Measurements

Various measures of cardiometabolic health were assessed in the eligible studies–energy metabolism, vascular function, incretins, inflammatory, oxidative stress and antioxidant status outcomes. Energy metabolism outcomes included glucose (GLU), insulin (INS), triglycerides (TG), total cholesterol (TC), high-density lipoprotein (HDL), low-density lipoprotein (LDL), non-esterified fatty acids (NEFA), adiponectin (APN) and apolipoprotein-B (Apo-B). Vascular function outcomes included augmentation index (AI) and pressure (AP), pulse wave velocity (PWV), systolic (SBP) and diastolic blood pressures (DBP), flow-mediated dilation (FMD), digital volume pulse-stiffness (DVP-SI) and -reflection indices (DVP-RI), pulse pressure (PP), heart rate (HR), large (LAEI) and small artery elasticity indices (SAEI), nitrites (NO_2_), nitrates (NO_3_), and nitric oxides (NO_x_). Incretins included pancreatic polypeptide (PPY), gastric inhibitory peptide (GIP), glucagon-like peptide 1 (GLP1), and peptide-tyrosine-tyrosine (PYY). Inflammatory outcomes included oxidized low-density lipoprotein (ox-LDL), interleukin-6 (IL-6), -17 (IL-17), -1b (IL-1b), and -10 (IL-10), tumor necrosis factor alpha (TNFα), C-reactive protein (CRP), chemerin (RARR2), and plasminogen activator inhibitor-1 (PAI-1). Oxidative stress and antioxidant status outcomes included total antioxidant capacity (TAC), F2-isoprostanes (F2-iso), uric acid (UA), ferric reducing ability of plasma (FRAP), 2,2′diphenyl-1-picrylhydrazyl radical (DPPH), oxygen radical absorbance capacity (ORAC), malondialdehyde (MDA), protein carbonyls (CO), and thiols (SH). Most studies (n = 17) had reported energy metabolism outcomes [12,16,17,18,19,20,21,23,26,27,28,29,30,31,32]; 11 studies had looked into inflammatory outcomes [16,17,18,19,20,21,23,24,26,27,28]; 8 studies had reported outcomes for oxidative stress and antioxidant status [12,16,17,21,22,25,28,29]; 5 studies had investigated outcomes of vascular function [12,16,18,27,28]; and 3 studies had assessed incretins [12,27,31].

### 3.3. Quality Assessment

Risk of bias for the 18 included articles were assessed according to the Cochrane Handbook for Systematic Reviews of Interventions [15]. Results of the assessment are summarized in Table 2. The designs for all 20 studies in 18 included articles were described as randomized, but 8 articles did not include details about random sequence generation [18,19,21,22,25,26,29,32]. Methods of allocation concealment were not provided by 13 articles [18,19,20,21,22,23,24,25,26,28,29,31,32]. The blinding of participants and personnel were considered to be unclear in 5 articles [18,21,24,25,26], while the blinding of outcome assessment was unclear in 7 articles [18,22,23,26,28,29,32]. All articles took a low risk of bias in selective reporting and incomplete outcome data. Two articles failed to indicate their source of funding [16,24]. Of the 18 eligible articles, 4 did not mention if there was any conflict of interest [16,22,26,32]. No articles reported a conflict of interest among authors.

### 3.4. Energy Metabolism

The results of 17 studies that reported outcomes for energy metabolism are summarized in Table 3. GLU (n = 15), INS (n = 13) and TG (n = 13) were the most common measures of energy metabolism. Other outcomes include TC (n = 4), HDL (n = 2), LDL (n = 2), NEFA (n = 3), APN (n = 1), and ApoB (n = 1). Seven of the 15 studies that assessed GLU had reported statistically significant treatment effects in the intervention group [12,18,20,23,27,30]. Of these 7 studies, 4 were among healthy participants [12,20,23,27], and 1 among the obese and diabetic [18], and 1 in prediabetics with insulin resistance [20]. All 7 studies had HC interventions, with anthocyanin doses ranging from 46 mg to 600 mg, and intervention periods of 2 h to 24 h. Of the 14 studies assessing the impact of intervention on INS, 5 reported a significant decrease in INS with anthocyanin intervention [12,17,19,20,30]. Four of the 5 studies were among the prediabetic, overweight and obese, and participants with insulin resistance [17,19,20,30]. One study was among healthy participants [12]. All 5 studies provided HC interventions, anthocyanin doses between 39 mg and 600 mg, with intervention periods of 2 h to 24 h. One study reported a significant decrease NEFA concentrations over a 2-h measurement in healthy participants, after a HC intervention with 600 mg of anthocyanins [27]. No other significant treatment effects were reported in the studies assessing the other outcomes for energy metabolism.

### 3.5. Vascular Function

Table 4 summarizes the 5 studies that reported outcomes for vascular function in response to anthocyanin interventions. SBP (n = 5) and DBP (n = 4) were the most common measures of vascular function. Only 1 of the 5 studies reported statistically significant differences in outcomes in the intervention group. This acute study reported significant increases in FMD, plasma NO_2_ and urinary NO_3_ responses over 4 h in participants at risk of CVD, after an HF intervention with 150 mg of anthocyanins [28]. No other significant treatment effects were reported in the 4 other studies that investigated the other outcomes for vascular function.

### 3.6. Incretins

Incretins were found to physiologically control appetite, improve satiety, and amplify insulin secretory mechanisms [33]. The inclusion of incretins as a potential measure for cardiometabolic health in this review was due to its potential role in modulating glycemic responses and body weight. The 3 studies that reported outcomes for incretins are presented in Table 5. Notably, all 3 studies reported statistically significant changes in incretins for the intervention group among healthy participants. Two studies had observed significant decreases in GIP responses [12,27]; one study each had noted significant decreases in PPY [31] and GLP-1 [12]. These changes in incretin responses were over 2 h, after HC interventions with anthocyanin doses between 401 mg and 600 mg. No significant treatment effects were reported for PYY.

### 3.7. Inflammation

Results for the 11 studies that evaluated the intervention effects on inflammatory outcomes are summarized in Table 6. Ox-LDL (n = 6) and IL-6 (n = 6) were the most common measures of inflammation among these studies. Of the 6 studies that investigated ox-LDL, 2 reported a significant decrease in ox-LDL responses after HC interventions over study periods of 3 to 6 h, with anthocyanins dosed between 88 mg to 161 mg [17,21]. Four of the 6 studies that assessed IL-6 observed a significant decrease in IL-6 responses [17,18,19,26]. Participants of these acute studies were given HC or HF interventions over study durations of 4 to 10 h, with anthocyanins dosed between 32 mg to 225 mg. Two acute studies each had observed significant decreases in TNF-α [18,26] and CRP [19,27]. One study reported significant declines in IL-17 [26]. No studies reported significant treatment effects in IL-10, PAI-1, and RARRE2.

### 3.8. Oxidative Stress and Antioxidant Status

Table 7 summarizes the 8 studies that assessed the effects of intervention on measures of oxidative stress and antioxidant status. ORAC (n = 3) was the most common measure among these studies. Two of these 3 studies reported significant increases in ORAC responses over study durations of 3 to 4 h, after a HC intervention with anthocyanin doses between 53 mg to 161 mg [21,22]. Significant treatment effects on outcomes of oxidative stress and antioxidant status were also noted in 4 other acute studies. One acute study each observed an increase in TAC [28] and significant declines in UA and SH [25], of which both provided HF interventions with 32 mg to 150 mg of anthocyanins. A study that incorporated 84.3 g of anthocyanin from a Chilean berry extract into the preparation of a HF meal had reported significant improvements in DPPH, MDA, and CO responses [29]. No other studies had highlighted significant differences in FRAP, TRAP, and F2-iso responses in the intervention group.

## 4. Discussion

This systematic literature review aims to synthesize findings from existing full-feeding nutritional intervention studies that assess the influence of anthocyanins on cardiometabolic health when it is consumed as part of a composite meal. Overall, the review of 18 articles comprising one chronic and 19 acute studies suggested that the consumption of anthocyanins with a composite meal may have a limited influence on measures of cardiometabolic health. From the five cardiometabolic outcomes assessed in this study, GLU (n = 15), INS (n = 13), GIP (n = 3), IL-6 (n = 6), ox-LDL (n = 6) and ORAC (n = 3) statuses were most commonly evaluated among included articles. Among these, anthocyanin intake improved GLU, GIP, IL-6 and ORAC as determined by area under curves (AUC) or postprandial concentrations. Seven of 15 studies, consisting of 140 of 278 participants (50.4%), reported improvements in GLU measurements with intervention. Five studies that assessed INS, comprising 98 of 269 participants (36.4%), had also observed post-intervention improvements in INS measurements. Consistent post-intervention data were observed in GIP, in which two of three studies (47 of 64 participants, or 73.4%) noted a reduction in GIP AUC or concentrations. Of 129 participants in six studies reporting IL-6 outcomes, 87 participants (67.4%) saw significant improvements after the anthocyanin intervention. On the contrary, only two of six studies assessing ox-LDL, making up 36 of 135 participants (26.7%), reported significant reductions after the intervention. Finally, two studies comprising 21 of 42 participants (50.0%) had reported significant elevations in postprandial ORAC levels with intervention. Although most of the included studies had reflected one or more successful outcomes from intervention, there was inconsistency in these findings and the potential effects presented in this review were mostly nonsignificant.

The included studies had dosed anthocyanins over a wide range of 32 mg to 600 mg, where a 100 g portion of blackberries contains approximately 170 mg anthocyanins [34]. Where a composite meal was employed, no dose-response relationships between anthocyanins and the reported outcomes were identified among the articles included in this review. Studies that used high doses of anthocyanins observed similar to no effect compared to those studies with lower doses that represented a more achievable intake. There were also no trends reflective of any anthocyanin source eliciting a more successful outcome than the others. The outcomes of RCTs that used anthocyanin-rich extracts as intervention were no different from those that used whole foods. Additionally, none of the included studies had utilized purified anthocyanins, or comparators that controlled for the presence of other bioactive compounds present naturally in the interventions. For the studies with successful outcomes, it thus remains inconclusive whether the anthocyanin itself or the other bioactive compounds resulted in improvements in cardiometabolic health. It is suggested that these other bioactive components could have contributed to the bioactivity threshold or synergistically influenced the investigated outcome [9].

Three composite meal interventions were represented in this review—the high-carbohydrate, high-fat, and both. All included studies were of controlled full-feeding designs to determine any potential causal relationship between the co-ingestion of anthocyanins with foods and physiological outcomes, while accounting for any differences in dietary intake on these outcomes [35]. Based on the included studies in this review, the results were not reflective of any meal composition contributing to a more successful outcome. It was unclear if meal compositions had influenced the intervention outcome. Moreover, it was plausible that due to the differences in food items used across the studies reviewed, the inconsistencies in food matrices and dietary components may have impacted the bioaccessibility of anthocyanins differently [36]. These factors could have confounded the bioavailability of anthocyanins, which is a precursor for bioactivity.

In this review, we had aimed to summarize both the long- and short-termed effects of co-ingesting anthocyanin with composite meals by only including full-feeding RCTs. However, the inclusion of only one 2-week study made it difficult to specifically evaluate the long-termed outcomes. Results in this review were thus largely focused on postprandial responses from the 19 acute studies, with study durations of 2 to 24 h. Given that people spend most of their waking hours in a non-fasting state, fasting blood measurements taken in many long-termed studies may not be fully indicative of the etiology behind cardiometabolic disorders [37]. The dysregulation of postprandial metabolism not only compromises the nutritional state of the individual, but also implicates their metabolic health [38]. The role of postprandial metabolic responses as predictive cardiometabolic risk determinants has been increasingly supported by accumulating clinical evidence [39,40,41,42,43], highlighting the relevance of diets and their metabolic consequences.

It has been suggested that the consumption of a diet rich in anthocyanins contributes to acute improvements in markers of cardiometabolic health. Numerous short-termed human studies, with interventions lasting up to a day, have investigated the effects of anthocyanin intake on cardiovascular and metabolic risk factors. The individual consumption of anthocyanin-rich foods or extracts were found to improve acute endothelial function (164 and 328 mg total anthocyanins) [44] and antioxidant status (2 mg total anthocyanins) [45], The ingestion of anthocyanin-rich extracts (152 mg total anthocyanins) had also significantly reduced 2-h postprandial glucose and insulin levels [46], an indication of improved glycemic control and insulin sensitivity. Additionally, the same study had observed significantly lower 4-h postprandial inflammatory responses. On the contrary, outcomes from the acute nutritional interventions represented in this review, with anthocyanin doses similar to the aforementioned studies, had reported no significant differences in postprandial glucose and insulin responses [16,21,28], vascular function [16,18,28], plasma antioxidant and oxidative stress levels [12,16,17], as well as inflammation [16,20,28]. Even among included composite meal studies with higher anthocyanin doses (>300 mg total anthocyanin), postprandial responses for glycemic [31], insulinemic [27,31], lipemic [27,32], vascular function [12,27], and oxidative stress [12] were not significantly improved after the acute intervention. Results from the included acute studies not only reflect a no dose-response relationship between the co-ingestion of anthocyanins with composite meals and cardiometabolic markers, but also a limited to non-significant influence on cardiometabolic health.

Findings from the present review suggest that the consumption of anthocyanins with food intake may not be effective in promoting cardiometabolic health. These findings contradict recent evidence of the preventive effects that anthocyanins have on the onset of metabolic and CVD. Epidemiological studies support the significant reductions in the relative risks of cardiometabolic disorders with diets rich in anthocyanin [3,4]. A meta-analysis of eight prospective cohorts reported a 15% decline in T2D risks after dietary anthocyanin and berry intakes [3], while another reported 11% reduced risks of CVD with anthocyanidin intakes in three prospective cohorts [6]. Other studies presented significant changes in lipid, inflammatory outcomes [8,47]. Articles included in these reviews comprise clinical studies with anthocyanins supplemented singularly or as part of a diet, and results are mostly representative of the chronic health outcomes from a general increase in anthocyanin consumption. While epidemiological evidence has strongly associated the chronic health benefits from anthocyanin consumption with mitigated risks of metabolic disease development, their results may be confounded by variables such as differences in dietary intake. Intervention studies have thus been conducted to confirm the health effects of anthocyanins in a more controlled design, but some of such studies still have not shown successful outcomes. For instance, nonsignificant changes in measures for energy metabolism were observed after the consumption of an anthocyanin-rich fruit juice or red raspberries [18,26]. The consumption of blackcurrant or strawberry extracts reported no significant treatment effects in vascular functions [12,16]. Inflammatory and oxidative stress indices were also not found to have changed significantly after the dosing of a strawberry extract [17]. The incongruence in health outcomes is usually attributed to dosing, study duration, inter-individual variability, and bioavailability. Additionally, it is suggested that matrix release kinetics and interactions with other dietary components within the food matrix have a substantial influence on the bioavailability of anthocyanins [13,48]. While demonstrating the clinical benefits of anthocyanin intake in different diets through intervention studies, there needs to be an adequate bioavailability before a beneficial effect is exerted [49]. The bioactivity of anthocyanins is highly dependent the food items it is consumed with. Many diets include multiple food items with simple to complex matrices and contain varying proportions of macronutrients. Variations in these nutrient and non-nutrient components alter the bioactivity of anthocyanins differently by influencing its bioaccessibility, uptake, and bioavailability [13]. 

In addition to investigating the attenuation of meal-induced postprandial metabolic responses by anthocyanin-rich interventions, one of the included studies had also attempted understand the influence of consumption timing on these outcomes [23]. This acute study delivered a relatively low dose of anthocyanins (49 mg) to overweight healthy adults at one of three different time points: 2 h before a meal, during a meal, and 2 h after a meal. In line with epidemiological evidence, findings from this study agreed that the consumption of dietary anthocyanins improve metabolic outcomes. However, significant improvements in metabolic responses were only reported for before- and after-meal interventions, where anthocyanin was ingested in the absence of other foods. Results from this study suggest that consumption timing as a variable may have a greater influence on cardiometabolic health than dosage. More importantly, it clarifies the influence of food matrices and dietary components in modulating the effects of anthocyanin on postprandial cardiometabolic responses.

Matrix effects, which are the physical and chemical interactions within food products, play a role in determining the release and the resulting bioactive properties of anthocyanins [50]. These matrix effects can be elucidated through in-vitro and in-vivo methodologies. While in-vitro simulated digestion methods may be employed to estimate the bioaccessibility of anthocyanins, in-vivo studies help to understand the degree of anthocyanin absorption by the human body post-ingestion, in order to estimate the potential health benefits of dietary anthocyanin intake. The bioaccessibility of anthocyanin has been investigated in several in-vitro studies. In a simulated starch digestion model, the co-digestion of raspberry extract with food had recovered 5% of the total phenols from the extract, which was lower than when the extract was digested alone [51]. Similarly, Sui et al. [2] had detected 3% of total anthocyanins in the digesta at the end of a 3-h simulated digestion of anthocyanin-fortified bread. Kan et al. [52] reported that the total anthocyanins released after the co-digestion of berry extract with bread were twice that of bread fortified with the same extract. In the same study, it was also noted that the total release of anthocyanins was different when co-digested separately with starch and gluten. The effects of food matrices were also observed in in-vivo studies, in which an increased plasma antioxidant capacity was observed after healthy participants ingested blueberries with water, but not with defatted milk [53]. A similar study also noted greater plasma radical-scavenging levels from blueberry intake in the absence of food, but not when blueberries were consumed with milk [54]. It was speculated that the differing antioxidant capacities in these in-vivo studies were a result of varying anthocyanin bioavailability from matrix effects. Matrix effects are also extended to other polyphenols, in which Chow, et al. [55] had reported greater bioavailability of green tea polyphenols when tea extract was consumed in a fasted state than in a postprandial state. Results from these studies have thus highlighted the influence of food matrices on polyphenol bioaccessibility, and consequently bioavailability and in-vivo health outcomes.

Dietary interventions in food-based human studies have different physical states, and are often introduced as a solid or a liquid, both of which differ in their matrices. Solid-based interventions require the breakdown of the food structure before gastric emptying, resulting in a lag phase. On the contrary, gastric emptying of liquid-based interventions are often immediate and at an exponential rate [56], contributing to a rapid polyphenol absorption from liquid foods [57]. Solid interventions often contain less water and more food constituents than solids in their matrix, from which may form conjugates with anthocyanins, thus limiting or delaying their release into the gastrointestinal tract. This difference in the matrix effect between solid and liquid foods is evident when Cassidy, et al. [58] reported a higher rate of polyphenol absorption from soymilk than solid soy foods. Additionally, a higher bioavailability of polyphenols was observed after the consumption of soy milk, relative to solid soybean food products [58,59]. 

Food-based interventions focus on the delivery of food and nutrients, with food matrices also differing in terms of their proximate content. Anthocyanin bioaccessibility and bioavailability may be influenced by matrix effects when co-delivered with foods rich in carbohydrates, proteins or fats. The interactions between dietary carbohydrates and anthocyanins have been confirmed in several in-vitro and human studies, but results have been contradictory. Anthocyanins were found to limit starch digestion and sugar uptake via interactions with amylase and α-glucosidase, as well as several glucose transporters, respectively [2,60]. On the other hand, improvements in anthocyanin bioavailability were reported with increased sugar intake, suggesting that anthocyanin acylation may have facilitated its uptake via sodium-glucose linked transporters [61,62]. In addition, Bub, et al. [63] suggested that higher glucose contents in food products may delay anthocyanin absorption, proposing a competitive interaction of glucose and anthocyanin with the sodium-dependent glucose cotransporter. The ingestion of grape juice, relative to red wine which had lower glucose content, had observed a slower absorption but higher bioavailability of malvidin-3-glucoside [63]. Similarly, Nielsen, et al. [64] had observed delayed peak plasma anthocyanin concentrations from the co-ingestion of blackcurrant juice with rice cakes. Conversely, the same study also reported no influence on total anthocyanin bioavailability, even when the rice cakes comprised 79% carbohydrates [64]. In the case of indigestible carbohydrates, it was suggested that they reduce the rate and extent of release of anthocyanins by forming soluble and insoluble polymer chains in the gastric lumen. While soluble fiber polymers limit the mixing of transport enzymes with polyphenolic substrates for absorption by increasing the viscosity of gastric fluids, insoluble fibers form complex structures which entraps the anthocyanin and limits accessibility [65].

There has been limited clinical evidence elucidating the release and absorption of anthocyanin in a protein-rich food matrix. However, the complexation of proteins and anthocyanins largely through covalent interactions have suggested the latter’s affinity for protein-rich food matrices [66,67]. A study reported significant reductions in plasma anthocyanin concentrations with strawberry milk consumption, suggesting possible interactions between strawberry anthocyanins and milk proteins that hindered anthocyanin bioavailability [68]. Through a gastrointestinal model, it was demonstrated that anthocyanins had improved accessibilities when complexed in a protein-rich matrix [69]. A similar observation was reported for protein-rich foods, through an in-vitro digestion model to study the bioaccessibility of pomegranate anthocyanins [70].

Dietary lipids may have limited influence on anthocyanins due to differences in hydrophilicity. Not many studies have assessed its effects on anthocyanin release from food matrices and absorption, and interactions between lipids and anthocyanins remain unclear. A clinical study reported an initial 2-h delay in pelargonidin bioavailability after the consumption of strawberries with cream, but total bioavailability over 24 h remained unchanged [71]. An investigation on blueberry anthocyanin bioaccessibility using a gastrointestinal model had concluded that lipid-rich food matrices did not impact the availability of anthocyanins [69]. Conversely, another digestion model reported that fatty acids significantly increased availability of anthocyanins [70].

We believe that this is the first systematic review on controlled full-feeding studies to specifically assess cardiometabolic outcomes from the co-ingestion of anthocyanins with a composite meal. Establishing causality between nutrients, diets, and disease risks is complex. Self-reported dietary assessment methods are often used in human nutritional research, but are often limited by measurement errors in data collection [72]. Controlled feeding studies are thus required to determine the causality between dietary intake and health outcomes. However, such studies are resource intensive, challenging, and may impose substantial participant burden. Consequently, this review is limited by the relatively small number of included controlled full-feeding studies, especially chronic studies, of which led to even smaller-sized subgroups as not all studies report each cardiometabolic marker. The lack of controlled feeding human nutritional studies in anthocyanin research is evident from this review, which impedes the elucidation of metabolic processes on how dietary intake influences the intended health benefits of anthocyanins. Another notable limitation of this review is the different test foods reported in the various studies, which restricts the generalizability of review findings. However, the strength of this review lies in that we only included full-feeding RCTs to limit any dietary factors that may confound the outcome. Additionally, this review summarizes the discrepancies between the successful outcomes of anthocyanin consumption reported in epidemiological studies and the negative results observed in intervention trials. Some trials attribute the lack of success to study design limitations including inadequate statistical power [18,21,24,28,31,32] or short intervention durations [16,18,24]. Another trial indicated that the use of healthy instead of unhealthy volunteers had contributed to the lack of significance in outcomes [16]. Other studies have also discussed inter-individual variability as a determinant for bioactivity [73,74]. As much as these factors need to be considered during the interpretation of findings and the conduct of future work, this review recognizes the need for further research into investigating the clinical significance of how food matrices can be modified to influence the bioaccessibility and bioavailability of anthocyanin. Knowledge in this area can be used to design future food or meal matrices to not only help improve the stability of bioactive compounds, but also enhance its release in the human body.

## 5. Conclusions

This review evaluated whether the co-delivery of anthocyanins with a composite meal was effective in improving markers for cardiometabolic disorders. Despite the heterogeneity in study interventions, consistent improvements were observed in GLU, GIP, IL-6 and ORAC responses. However, the overall findings from this review do not fully explain the efficacy of co-ingesting anthocyanins with other foods on improving cardiometabolic health. There is a need for more RCTs of full-feeding designs to evaluate the chronic effects of anthocyanin on metabolic profiles. In order to bridge the gap between the dietary consumption of anthocyanins and its metabolic consequences, further studies should also not only characterize the postprandial responses to foods of different matrices, but also evaluate the impact of different food matrices on the bioaccessibility and bioavailability of anthocyanin.

## Figures and Tables

**Table 1:** Characteristics of 20 included nutritional intervention studies on anthocyanin intake and cardiometabolic health in controlled feeding trials.

Reference	Country	Study Design	Subjects; M/F	Mean Age (SD)	Health Status	Intervention Length	%C/P/F; Meal Type; Meal Components	Anthocyanin Source	Dose	Control	Markers
Murkovic et al., 2004 [32]	Austria	R/C/DB/P	34; 20/14	Elderberry: 30 (6) Control: 28 (4)	Healthy	2 weeks	Breakfast: -; HC; coffee, bread rolls, butter, jam Lunch & dinner: 45/20/35; HC/MF; - (meals prepared at a local restaurant, standardized for nutrient distribution)	Gel capsules of spray-dried elderberry juice	300 mg	Placebo gel capsules	TG, TC, HDL, LDL
		R/C/DB/X	6; -	29 (5.1)	Healthy	6 h	18/11/71; HC; -	Gel capsules of spray-dried elderberry juice	400 mg	Placebo gel capsules	TG, TC, Apo-B
Prior et al., 2007 [16] (Study #5)	USA	R/C/X	6; 0/6	46.3 (5.6)	Healthy	4 h	80/7/13; HC; coconut milk, coffee creamer, ProMod protein powder, sugar, water, Polycose powder	Freeze-dried grape powder	53 mg	No freeze-dried grape powder	ORAC
Edirisinghe et al., 2011 [17]	USA	R/C/SB/X	24; 10/14	50.9 (15)	Overweight	6 h	56/15/29; HC; bagel, cream cheese, margarine, hard-boiled egg, cantaloupe, whole milk	Beverage containing freeze-dried strawberry powder	39 mg	Calorie-matched strawberry-flavored beverage with no extract	GLU, INS, IL-6, IL1-b, TNF-⍺, CRP, PAI-1
Serafini et al., 2011 [24]	Italy	R/C/DB/X	14; 12/2	45.1 (8.6)	Overweight	8 h	30/15/55; HF; fried potatoes, fried eggs, cheese, white bread	Mixture of pineapple, plum, and blackcurrant juices	32 mg	Calorie-matched placebo beverage	GLU, INS, TG, TC, APN, IL-6, IL-17, TNF-⍺, RARR2
Blacker et al., 2013 [18]	USA	R/C/X	15; -	22.2	Healthy	3 h	-; HC; corn flakes, milk	Freeze-dried blueberry powder dissolved in water	75, 161 mg	Calorie-matched control powder dissolved in water	GLU, ox-LDL, UA, ORAC
Hidalgo et al., 2014 [29]	Chile	R/C/DB/X	10; -	-	-	3 h	-; HC; boiled rice	Delphinol^®^ tablet dissolved in water	70 mg	Commercial instant powdered sugar-free berry juice with artificial coloring	GLU, INS
Miglio et al., 2014 [25]	Italy	R/C/DB/X	14; -	45 (9)	Overweight	24 h	29/15/55; HF; fried potatoes, fried eggs, cheese, white bread	Mixture of pineapple, plum, and blackcurrant juices	32 mg	Calorie-matched placebo beverage	F2-iso, UA, thiols, TRAP
Di Renzo et al., 2015 [26]	Italy	R/C/X	24; -	31 (5,9)	Healthy	3 h	28/18/54; HF; McDonald’s meal comprising the Big Tasty Bacon burger and French Fries	Red wine	541 mg/kg berry	Negative control: red wine only Positive control: test meal only	ox-LDL
Castro-Acosta et al., 2016 [12]	UK	R/C/DB/X	22; 13/9	45.4 (13.7)	Healthy	2 h	92/-/-; HC; Sliced white bread, apricot jam	Beverage containing blackcurrant extract	150, 300, 600 mg	Calorie-matched beverage with no extract	BP, DVP-SI, DVP-RI, GLU, INS, TG, GIP, GLP-1, NEFA, F2-iso
Huang et al., 2016 [19]	USA	R/C/SB/P	24; 16/8	Strawberry: 25 (4) Control: 27 (4)	Healthy	10 h	46/10/44; HC/HF; croissant, apple jelly, butter, frosted flake cereal, milk, breakfast sausage	Beverage containing freeze-dried strawberry powder	49 mg	Calorie-matched control beverage	GLU, INS, TG, ox-LDL, IL-6
Park et al., 2016 [20]	USA	R/C/SB/X	21, 5/16	39.8 (13.8)	Obese with insulin resistance	6 h	61/15/24, HC	Beverage containing freeze-dried strawberry powder	42, 88, 155 mg	Calorie-matched beverage with no powder	Glu, Ins, TG, ox-LDL, IL-6
Castro-Acosta et al., 2017 [27]	UK	R/C/DB/X	25; 20/5	32.3 (14.4)	Healthy	2 h	82/-/-; HC; white bread, apricot jam	Beverage containing blackcurrant and apple extracts	600 mg	Calorie-matched beverage with no extract	BP, DVP-SI, DVP-RI, GLU, INS, TG, GIP, NEFA, CRP
Richter et al., 2017 [21]	USA	R/C/SB/X	30; 17/13	28 (2)	Healthy, overweight, and obese	4 h	42/13/45; HF; cheese blintzes, heavy whipped cream, strawberry-flavored syrup, hard-boiled egg, bacon	Freeze-dried strawberry powder	163 mg	Calorie-matched placebo powder with strawberry flavoring	AI, AP, PWV, BP, GLU, INS, TG, MDA, ox-LDL
Urquiaga et al., 2017 [30]	Chile	R/C/X	9; 9/0	20	Healthy	6 h	-; HF; ground turkey leg meat burger	Chilean berry concentrate	Beverage: 90 mg; burger: 84.3 mg	Water	GLU, TG, FRAP, DPPH, CO, MDA,
Abubakar et al., 2019 [28]	UK	R/C/SB/X	25; 25/0	49 (2)	1–10% CVD risk in 10 years, as determined by QRISK 2	4 h	37/4/59; HF; buttered croissant, butter, honey,	Hibiscus beverage	150 mg	Water	AI, BP, HR, PP, FMD, NO2, NO3, NOx, GLU, INS, TG, NEFA, CRP, TAC
Schell et al., 2019 [22]	USA	R/C/X	25; 5/20	54 (4.2)	Obese and diabetic	4 h	23/13/64; HF; scrambled eggs, butter, hash brown potatoes, buttermilk biscuits, sausage patty	Frozen red raspberries	225 mg	Calorie and carbohydrate-matched control	BP, LAEI, SAEI, GLU, INS, TG, TC, HDL, LDL, IL-6, IL-1b, TNF-⍺, CRP, PAI-1,
Stote et al., 2019 [31]	Canada	R/C/X	17; 4/13	47 (15)	Healthy	2 h	76/5/19; HC; waffles, maple syrup	Whole blueberries	401 mg	Calorie-matched placebo gel	GLU, INS, PPY, GIP, GLP-1, PYY
Xiao et al., 2019 [23]	USA	R/C/SB/X	21; 12/9	All: 34 (12) PreDM + IR: 38 (13)	PreDM + IR	24 h	57/9/34; HC/MF; bagel, cream cheese, butter, cereal, whole milk	Frozen red raspberries	73, 146 mg	No raspberries	GLU, INS, TG, ox-LDL, IL-6, IL-10
		R/C/SB/X	11; 5/6	All: 34 (12) Healthy: 28 (6)	Healthy	24 h	57/9/34; HC/MF; bagel, cream cheese, butter, cereal, whole milk	Frozen red raspberries	73, 146 mg	No raspberries	GLU, INS, TG

Abbreviations for (1) study characteristics: R, randomised; C, controlled; SB, single-blinded; DB, double-blinded; X, crossover; P, parallel; -, not reported; %C/P/F, percentage calories from carbohydrates/proteins/fats; PreDM, prediabetic (type 2); IR, insulin resistance; CVD, cardiovascular disease; HC, high-carbohydrate; HF, high-fat; (2) vascular markers: AI, augmentation index; AP, augmentation pressure; PWV, pulse wave velocity; SBP, systolic blood pressure; DBP, diastolic blood pressure; FMD, flow mediated dilation; DVP-SI, digital volume pulse—stiffness index; DVP-RI, digital volume pulse—reflection index; LAEI, large artery elasticity index; SAEI, small artery elasticity index; PP, pulse pressure; NO_2_, nitrites; NO_3_, nitrates; NO_x_, nitric oxides; (3) energy metabolism markers: GLU, blood glucose; INS, serum insulin; TG, triglycerides; TC, total cholesterol; HDL, high-density lipoprotein; LDL, low-density lipoprotein; NEFA, non-esterified fatty acid; APN, adiponectin; Apo-B, apolipoprotein-B; (4) incretin markers: PPY, pancreatic polypeptide; GIP, gastric inhibitory peptide; GLP-1 glucagon-like peptide 1; peptide-tyrosine-tyrosine; (5) inflammatory markers: ox-LDL, oxidized low-density lipoprotein; IL-6, interleukin 6; il-17, interleukin 17; IL-1b, interleukin 1b; IL-10, interleukin 10; TNF-⍺, tumor necrosis factor alpha; CRP, c-reactive protein; PAI-1, plasminogen activator inhibitor 1; RARR2, chemerin; (6) oxidative stress and antioxidant status markers: TAC, total antioxidant capacity; F2-iso, F2 isoprostanes; UA, uric acid; FRAP, ferric reducing ability of plasma; TRAP, total peroxyl radical-trapping potential; DPPH, 2,2′-diphenyl-1-picrylhydrazyl radical; ORAC, oxygen radical absorbance capacity; MDA, malondialdehyde; CO, protein carbonyls; SH, thiols.

**Table 2:** Risk of bias assessment for included studies.

Reference	Random Sequence Generation	Allocation Concealment	Selective Reporting	Blinding (Participants and Personnel)	Blinding (Outcome Assessment)	Incomplete Outcome Data
Murkovic et al., 2004 [32]	U	U	L	L	U	L
Prior et al., 2007 [16]	U	U	L	L	U	L
Edirisinghe et al., 2011 [17]	U	U	L	L	L	L
Serafini et al., 2011 [24]	U	U	L	U	U	L
Blacker et al., 2013 [18]	U	U	L	U	L	L
Hidalgo et al., 2014 [29]	L	L	L	L	L	L
Miglio et al., 2014 [25]	U	U	L	U	L	L
Di Renzo et al., 2015 [26]	L	U	L	U	L	L
Castro-Acosta et al., 2016 [12]	L	L	L	L	L	L
Huang et al., 2016 [19]	L	U	L	L	U	L
Park et al., 2016 [20]	L	L	L	L	L	L
Castro-Acosta et al., 2017 [27]	L	L	L	L	L	L
Richter et al., 2017 [21]	L	L	L	L	L	L
Urquiaga et al., 2017 [30]	U	U	L	L	U	L
Abubakar et al., 2019 [28]	L	U	L	L	U	L
Schell et al., 2019 [22]	U	U	L	U	U	L
Stote et al., 2019 [31]	L	U	L	L	L	L
Xiao et al., 2019 [23]	L	U	L	L	L	L

**Table 3:** Effects of anthocyanin intake in composite meals on markers of energy metabolism.

Reference	Markers								
	GLU	INS	TG	TC	HDL	LDL	NEFA	APN	Apo-B
Murkovic et al., 2004 [32] (2 weeks)	-	-	NS	NS	-	-	-	-	NS
Murkovic et al., 2004 [32] (6 h)	-	-	NS	NS	NS	NS	-	-	-
Edirisinghe et al., 2011 [17]	NS	↓	-	-	-	-	-	-	-
Serafini et al., 2011 [24]	NS	NS	NS	NS	-	-	-	NS	-
Blacker et al., 2013 [18]	NS	-	-	-	-	-	-	-	-
Hidalgo et al., 2014 [29]	↓ conc. at 60 and 90 min	↓ conc. at 60 min	-	-	-	-	-	-	-
Castro-Acosta et al., 2016 [12]	↓ IAUC 0–30 min (600 mg) ↓ conc. 10–30 min and 75 min (600 mg)	↓ IAUC 0–30 min (600 mg) ↓ conc. 10–30 min and 75 min (600 mg)	NS	-	-	-	NS	-	-
Huang et al., 2016 [19]	↓ AUC 0–10 h (before- and after-meal groups only)	NS	NS	-	-	-	-	-	-
Park et al., 2016 [20]	NS	↓ peak insulin (155 mg only ↓ conc. 0–6 h (155 mg)	NS	-	-	-	-	-	-
Castro-Acosta et al., 2017 [27]	↓ IAUC 0–30 min only	NS	NS	-	-	-	↓ conc. 60–90 min only	-	-
Richter et al., 2017 [21]	NS	NS	NS	-	-	-	-	-	-
Urquiaga et al., 2017 [30]	NS	-	NS	-	-	-	-	-	-
Abubakar et al., 2019 [28]	NS	NS	NS	-	-	-	NS	-	-
Schell et al., 2019 [22]	↓ AUC ↓ conc at 4 h	NS	NS	NS	NS	NS	-	-	-
Stote et al., 2019 [31]	NS	NS	-	-	-	-	-	-	-
Xiao et al., 2019 [23] (healthy)	↑ conc. at 2 h only	NS	NS	-	-	-	-	-	-
Xiao et al., 2019 [23] (PreDM)	↓ peak glucose (146 mg)	↓	NS	-	-	-	-	-	-

Abbreviations: GLU, blood glucose; INS, serum insulin; TG, triglycerides; TC, total cholesterol; HDL, high-density lipoprotein; LDL, low-density lipoprotein; NEFA, non-esterified fatty acid; APN, adiponectin; Apo-B, apolipoprotein-B; -, not reported.

**Table 4:** Effects of anthocyanin intake in composite meals on markers for vascular functions.

Reference	Markers														
	SBP	DBP	DVP-SI	DVP-RI	AI	AP	PWV	HR	PP	FMD	LAEI	SAEI	NO2	NO3	NOx
Castro-Acosta et al., 2016 [12]	NS	NS	NS	NS	-	-	-	-	-	-	-	-	-	-	-
Castro-Acosta et al., 2017 [27]	NS	NS	NS	NS	-	-	-	-	-	-	-	-	-	-	-
Richter et al., 2017 [21]	NS	-	-	-	NS	NS	NS	-	-	-	-	-	-	-	-
Abubakar et al., 2019 [28]	NS	NS	-	-	NS	-	-	NS	NS	↑ %FMD 0–4 h	-	-	↑ plasma conc. 0–4 h	↑ urinary conc. at 4 h only	NS
Schell et al., 2019 [22]	NS	NS	-	-	-	-	-	-	-	-	NS	NS	-	-	-

Abbreviations: AI, augmentation index; AP, augmentation pressure; PWV, pulse wave velocity; SBP, systolic blood pressure; DBP, diastolic blood pressure; FMD, flow mediated dilation; DVP-SI, digital volume pulse—stiffness index; DVP-RI, digital volume pulse—reflection index; LAEI, large artery elasticity index; SAEI, small artery elasticity index; PP, pulse pressure; NO_2_, nitrites; NO_3_, nitrates; NO_x_, nitric oxides; -, not reported.

**Table 5:** Effects of anthocyanin intake in composite meals on incretin hormones.

Reference	Markers			
	PPY	GIP	GLP-1	PYY
Castro-Acosta et al., 2016 [12]	-	↓ IAUC 0–2 h (600 mg) ↓ conc. 0–2 h (600 mg) ↓ peak GIP (600 mg)	↓ conc. at 90 min only (600 mg)	-
Castro-Acosta et al., 2017 [27]	-	↓ IAUC 0–30 min	-	-
Stote et al., 2019 [31]	↑ conc. 30–120 min	NS	NS	NS

Abbreviations: PPY, pancreatic polypeptide; GIP, gastric inhibitory peptide; GLP-1 glucagon-like peptide 1; peptide-tyrosine-tyrosine; -, not reported.

**Table 6:** Effects of anthocyanin intake in composite meals on inflammatory markers.

Reference	Markers								
	ox-LDL	IL-6	IL-17	IL-1b	IL-10	TNF-⍺	CRP	PAI-1	RARR2
Edirisinghe et al., 2011 [17]	-	↓ conc. at 6 h only	-	NS	-	NS	↓ mean 6 h-conc.	NS	-
Serafini et al., 2011 [24]	-	↓ conc. 30–120 min	↓ conc. at 4 h and 8 h	-	-	↓ conc. 0.5–8 h	-	-	NS
Blacker et al., 2013 [18]	↓ AUC 0–3 h	-	-	-	-	-	-	-	-
Di Renzo et al., 2015 [26]	NS	-	-	-	-	-	-	-	-
Huang et al., 2016 [19]	NS	↓ AUC 0–10 h (before- and after-meal groups only)	-	-	-	-	-	-	-
Park et al., 2016 [20]	↓ conc. 0–6 h (88mg)	NS	-	-	-	-	-	-	-
Castro-Acosta et al., 2017 [27]	-	-	-	-	-	-	↓ IAUC 0–2 h	-	-
Richter et al., 2017 [21]	NS	-	-	-	-	-	-	-	-
Abubakar et al., 2019 [28]	-	-	-	-	-	-	NS	-	-
Schell et al., 2019 [22]	-	↓ conc. at 4 h only	-	NS	-	↓ conc. at 4 h only	NS	NS	-
Xiao et al., 2019 [23] (PreDM)	NS	NS	-	-	NS	-	-	-	-

Abbreviations: ox-LDL, oxidized low-density lipoprotein; IL-6, interleukin 6; il-17, interleukin 17; IL-1b, interleukin 1b; IL-10, interleukin 10; TNF-⍺, tumor necrosis factor alpha; CRP, c-reactive protein; PAI-1, plasminogen activator inhibitor 1; RARR2, chemerin; -, not reported.

**Table 7:** Effects of anthocyanin intake in composite meals on markers for oxidative stress and antioxidant status.

Reference	Markers									
	ORAC	FRAP	TRAP	DPPH	TAC	MDA	F2-iso	UA	SH	CO
Prior et al., 2007 [16]	↑ AUC 0–4 h	-	-	-	-	-	-	-	-	-
Blacker et al., 2013 [18]	↑ AUC 0–1 h	-	-	-	-	-	-	NS	-	-
Miglio et al., 2014 [25]	-	-	NS	-	-	-	NS	↓ conc. at 8 h only	↓ conc. at 2 h, 4 h and 8 h only	-
Castro-Acosta et al., 2016 [12]	-	-	-	-	-	-	NS	-	-	-
Park et al., 2016 [20]	NS	-	-	-	-	-	-	-	-	-
Richter et al., 2017 [21]	-	-	-	-	-	NS	-	-	-	-
Urquiaga et al., 2017 [30]	-	NS	-	↑ AUC 0–6 h	-	↓ AUC 0–6 h	-	-	-	↓ AUC 0–6 h
Abubakar et al., 2019 [28]	-	-	-	-	↑ AUC 0–2 h	-	-	-	-	-

Abbreviations: TAC, total antioxidant capacity; F2-iso, F2 isoprostanes; UA, uric acid; FRAP, ferric reducing ability of plasma; TRAP, total peroxyl radical-trapping potential; DPPH, 2,2′-diphenyl-1-picrylhydrazyl radical; ORAC, oxygen radical absorbance capacity; MDA, malondialdehyde; CO, protein carbonyls; SH, thiols; -, not reported.

## Supplementary Materials

The following are available online at https://www.mdpi.com/2072-6643/12/12/3781/s1, Table S1: Full electronic search strategy.
