# Supplementary material for: Effects of Anthocyanins in Composite Meals on Cardiometabolic Outcomes—A Systematic Review of Randomized Controlled Feeding Trials
Source: Nutrients. 2020 Dec 9;12(12):3781. doi: 10.3390/nu12123781 (PMC7763924; doi:10.3390/nu12123781)
Supplement: Supplementary file 1 [file nutrients-12-03781-s001.pdf]

Table S1. Full electronic search strategy.

**PubMed Search Strategy**		
**No.**	**Query**	**Results**
#1	("Anthocyanins"[MeSH]) OR (anthocyanin*[tiab] OR anthocyanidin*[tiab] OR leucoanthocyanidin*[tiab] OR "anthocyanin glycoside*"[tiab] OR "anthocyanine glycoside*"[tiab] OR "cassis anthocyanoside*"[tiab] OR cyanidin*[tiab] OR "cyanidin 3 glucoside*"[tiab] OR "cyanidin 3-O-glucoside*"[tiab] OR delphinidin*[tiab] OR "delphinidin 3 glucoside*"[tiab] OR "delphinidin 3-O-glucoside*"[tiab] OR pelargonidin* OR "pelargonidin 3 glucoside*"[tiab] OR "pelargonidin 3-O-glucoside*"[tiab] OR peonidin*[tiab] OR "peonidin 3 glucoside*"[tiab] OR "peonidin 3-O-glucoside*"[tiab] OR petunidin*[tiab] OR "petunidin 3 glucoside*"[tiab] OR "petunidin 3-O-glucoside*"[tiab] OR malvidin*[tiab] OR "malvidin 3 glucoside*"[tiab] OR "malvidin 3-O-glucoside*"[tiab] OR "anthocyanin intake*"[tiab] OR "anthocyanin consum*"[tiab] OR berry[tiab] OR berries[tiab] OR chokeberr*[tiab] OR aronia[tiab] OR melanocarpa[tiab] OR aubergine*[tiab] OR brinjal*[tiab] OR eggplant*[tiab] OR "solanum melongena*"[tiab] OR "Guinea squash"[tiab] OR "Solanum insanum"[tiab] OR "black currant*"[tiab] OR "Ribes nigrum"[tiab] OR blueberr*[tiab] OR "Vaccinium corymbosum"[tiab] OR "Vaccinium cyanococcus"[tiab] OR "blood orange*"[tiab] OR cherry[tiab] OR cherries[tiab] OR "Cerasus vulgaris"[tiab] OR "Prunus cerasus"[tiab] OR "Prunus avium"[tiab] OR grape*[tiab] OR rhubarb[tiab] OR "rheum rhabarbarum"[tiab] OR strawberr*[tiab] OR "fragaria vesca"[tiab] OR "Fragaria ananassa"[tiab] OR blackberr*[tiab] OR raspberr*[tiab] OR "rubus glaucus"[tiab] OR "Rubus fruticosus"[tiab] OR plum*[tiab] OR "red cabbage*" OR "purple cabbage*"[tiab] OR "Brassica oleracea var capitata f rubra"[tiab] OR "red wine"[tiab] OR cranberr*[tiab] OR "vaccinium macrocarpon*"[tiab] OR elderberr*[tiab] OR "sambucus Canadensis"[tiab] OR bilberr*[tiab] OR "vaccinium myrtillus"[tiab] OR whortleberr*[tiab])	64969
#2	("Cardiovascular disease"[MeSH] OR "Glucose metabolism disorders"[MeSH] OR "Lipid metabolism disorders"[MeSH] OR "Metabolic syndrome"[MeSH] OR "Inflammation"[MeSH] OR "Oxidative stress"[MeSH] OR "Antioxidant"[MeSH]) OR ("cardiovascular disease*"[tiab] OR "atherosclerotic cardiovascular disease*"[tiab] OR "cardiovascular risk*"[tiab] OR "cardio-vascular disease*"[tiab] OR angiocardiopath*[tiab] OR "angiocardiovascular disease*"[tiab] OR "cardiovascular complication*"[tiab] OR "cardiovascular disorder*"[tiab] OR "cardiovascular disturbance*"[tiab] OR "cardiovascular lesion*"[tiab] OR "cardiovascular syndrome*"[tiab] OR "cardiovascular vegetative disorder*"[tiab] OR "cardiovascular complication*"[tiab] OR "major adverse cardiovascular event*"[tiab] OR "glucose metabolism disorder*"[tiab] OR "glucose metabolic disorder*"[tiab] OR "biotin responsive multiple carboxylase deficienc*"[tiab] OR "combined carboxylase deficienc*"[tiab] OR "glucose metabolism disorder*"[tiab] OR "holocarboxylase deficienc*"[tiab] OR "holocarboxylase synthetase deficienc*"[tiab] OR "mckusick 25327"[tiab] OR "multiple carboxylase deficienc*"[tiab] OR "multiple carboxylase deficienc*"[tiab] OR "lipid metabolism disorder*"[tiab] OR "lipid metabolic disorder*"[tiab] OR "nutritional disorder*"[tiab] OR "metabolic syndrome*"[tiab] OR "metabolic syndrome X"[tiab] OR "insulin resistance syndrome X"[tiab] OR "metabolic X syndrome"[tiab] OR "dysmetabolic syndrome X"[tiab] OR "reaven syndrome X"[tiab] OR "metabolic cardiovascular syndrome*"[tiab] OR "body function disorder*"[tiab] OR "pathologic process*"[tiab] OR "pathological condition*"[tiab] OR "symptoms and general pathology"[tiab] OR inflammat*[tiab] OR "inflammation reaction*"[tiab] OR "inflammatory reaction*"[tiab] OR "inflammation response*"[tiab] OR "inflammatory response*"[tiab] OR "inflammatory condition*"[tiab] OR "inflammatory lesion*"[tiab] OR "inflammatory process*"[tiab] OR "inflammatory syndrome*"[tiab] OR "inflammatory activ*"[tiab] OR "inflammatory disease*"[tiab] OR "systemic inflammat*"[tiab] OR "acute inflammat*"[tiab] OR "chronic inflammat*"[tiab] OR serositis[tiab] OR "anti-inflammat*"[tiab] OR "anti-inflammatory effect*"[tiab] OR "anti-inflammatory activ*"[tiab] OR "antiinflammat*"[tiab] OR "antiinflammatory activ*"[tiab] OR "oxidative stress*"[tiab] OR "oxidative capacit*"[tiab] OR "oxidative damag*"[tiab] OR "oxidative balance*"[tiab] OR "oxidative stress-induced damag*"[tiab] OR "oxidative stress induced damag*"[tiab] OR "oxidation reduction stat*"[tiab] OR "oxidative DNA damag*"[tiab] OR damag*[tiab] OR "oxidant stress*"[tiab] OR antioxida* OR anti-oxida*[tiab] OR "anti oxida*"[tiab] OR "antioxidant effect*"[tiab] OR "anti-oxidant effect*"[tiab] OR "anti oxidant effect*"[tiab] OR "antioxidative effect*"[tiab] OR "anti-oxidative effect*"[tiab] OR "anti oxidative effect*"[tiab] OR "antioxidant capacit*"[tiab] OR "anti-oxidant capacit*"[tiab] OR "anti oxidant capacit*"[tiab] OR "antioxidative capacit*"[tiab] OR "anti-oxidative capacit*"[tiab] OR "anti oxidative capacit*"[tiab] OR "antioxidant activ*"[tiab] OR "anti-oxidant activ*"[tiab] OR "anti oxidant activ*"[tiab] OR "antioxidative activ*"[tiab] OR "anti-oxidative activ*"[tiab] OR "anti oxidative activ*"[tiab] OR "antioxidant response element*"[tiab] OR "anti oxidant response element*"[tiab] OR "anti-oxidant response element*"[tiab] OR "antioxidant assay*"[tiab] OR "anti-oxidant assay*"[tiab] OR "anti oxidant assay*"[tiab] OR "ferric reducing ability of plasma"[tiab] OR "antioxidant capacity assay*"[tiab] OR "antioxidant activity assay*"[tiab] OR "antioxidative assay*"[tiab] OR "oxygen radical absorbance capacity assay*"[tiab] OR "cell-based antioxidant protection assay*"[tiab] OR "thiobarbituric acid reactive substance assay*"[tiab] OR "lipid peroxidation assay*"[tiab] OR "free radical scavenging assay*"[tiab])	2661344

#3	(("randomized controlled trial"[pt] OR "controlled clinical trial"[pt] OR randomized[tiab] OR placebo[tiab] OR "clinical trials as topic"[MeSH: noexp] OR randomly[tiab] OR trial[ti]) NOT (animals[mh] NOT humans[mh]))	1153512
#4	#1 AND #2 AND #3	952

**Embase Search Strategy**		
**No.**	**Query**	**Results**
#1	("Anthocyanin"/exp) OR (anthocyanin*:ti,ab OR anthocyanidin*:ti,ab OR leucoanthocyanidin*:ti,ab OR "anthocyanin glycoside*":ti,ab OR "anthocyanine glycoside*":ti,ab OR "cassis anthocyanoside*":ti,ab OR cyanidin*:ti,ab OR "cyanidin 3 glucoside*":ti,ab OR "cyanidin 3-O-glucoside*":ti,ab OR delphinidin*:ti,ab OR "delphinidin 3 glucoside*":ti,ab OR "delphinidin 3-O-glucoside*":ti,ab OR pelargonidin* OR "pelargonidin 3 glucoside*":ti,ab OR "pelargonidin 3-O-glucoside*":ti,ab OR peonidin*:ti,ab OR "peonidin 3 glucoside*":ti,ab OR "peonidin 3-O-glucoside*":ti,ab OR petunidin*:ti,ab OR "petunidin 3 glucoside*":ti,ab OR "petunidin 3-O-glucoside*":ti,ab OR malvidin*:ti,ab OR "malvidin 3 glucoside*":ti,ab OR "malvidin 3-O-glucoside*":ti,ab OR "anthocyanin intake*":ti,ab OR "anthocyanin consum*":ti,ab OR berry:ti,ab OR berries:ti,ab OR chokeberr*:ti,ab OR aronia:ti,ab OR melanocarpa:ti,ab OR aubergine*:ti,ab OR brinjal*:ti,ab OR eggplant*:ti,ab OR "solanum melongena*":ti,ab OR "Guinea squash":ti,ab OR "Solanum insanum":ti,ab OR "black currant*":ti,ab OR "Ribes nigrum":ti,ab OR blueberr*:ti,ab OR "Vaccinium corymbosum":ti,ab OR "Vaccinium cyanococcus":ti,ab OR "blood orange*":ti,ab OR cherry:ti,ab OR cherries:ti,ab OR "Cerasus vulgaris":ti,ab OR "Prunus cerasus":ti,ab OR "Prunus avium":ti,ab OR grape*:ti,ab OR rhubarb:ti,ab OR "rheum rhabarbarum":ti,ab OR strawberr*:ti,ab OR "fragaria vesca":ti,ab OR "Fragaria ananassa":ti,ab OR blackberr*:ti,ab OR raspberr*:ti,ab OR "rubus glaucus":ti,ab OR "Rubus fruticosus":ti,ab OR plum*:ti,ab OR "red cabbage*" OR "purple cabbage*":ti,ab OR "Brassica oleracea var capitata f rubra":ti,ab OR "red wine":ti,ab OR cranberr*:ti,ab OR "vaccinium macrocarpon*":ti,ab OR elderberr*:ti,ab OR "sambucus Canadensis":ti,ab OR bilberr*:ti,ab OR "vaccinium myrtillus":ti,ab OR whortleberr*:ti,ab)	76489
#2	("Cardiovascular disease"/exp OR "Disorders of carbohydrate metabolism"/exp OR "Disorders of lipid and lipoprotein metabolism"/exp OR "Physical disease by body function"/exp OR "Inflammation"/exp OR "Oxidative stress"/exp OR "Antioxidant activity"/exp OR "Antioxidant assay"/exp OR "Antioxidant responsive element"/exp) OR ("cardiovascular disease*":ti,ab OR "atherosclerotic cardiovascular disease*":ti,ab OR "cardiovascular risk*":ti,ab OR "cardio-vascular disease*":ti,ab OR angiocardiopath*:ti,ab OR "angiocardiovascular disease*":ti,ab OR "cardiovascular complication*":ti,ab OR "cardiovascular disorder*":ti,ab OR "cardiovascular disturbance*":ti,ab OR "cardiovascular lesion*":ti,ab OR "cardiovascular syndrome*":ti,ab OR "cardiovascular vegetative disorder*":ti,ab OR "cardiovascular complication*":ti,ab OR "major adverse cardiovascular event*":ti,ab OR "glucose metabolism disorder*":ti,ab OR "glucose metabolic disorder*":ti,ab OR "biotin responsive multiple carboxylase deficienc*":ti,ab OR "combined carboxylase deficienc*":ti,ab OR "glucose metabolism disorder*":ti,ab OR "holocarboxylase deficienc*":ti,ab OR "holocarboxylase synthetase deficienc*":ti,ab OR "mckusick 25327":ti,ab OR "multiple carboxylase deficienc*":ti,ab OR "multiple carboxylase deficienc*":ti,ab OR "lipid metabolism disorder*":ti,ab OR "lipid metabolic disorder*":ti,ab OR "nutritional disorder*":ti,ab OR "metabolic syndrome*":ti,ab OR "metabolic syndrome X":ti,ab OR "insulin resistance syndrome X":ti,ab OR "metabolic X syndrome":ti,ab OR "dysmetabolic syndrome X":ti,ab OR "reaven syndrome X":ti,ab OR "metabolic cardiovascular syndrome*":ti,ab OR "body function disorder*":ti,ab OR "pathologic process*":ti,ab OR "pathological condition*":ti,ab OR "symptoms and general pathology":ti,ab OR inflammat*:ti,ab OR "inflammation reaction*":ti,ab OR "inflammatory reaction*":ti,ab OR "inflammation response*":ti,ab OR "inflammatory response*":ti,ab OR "inflammatory condition*":ti,ab OR "inflammatory lesion*":ti,ab OR "inflammatory process*":ti,ab OR "inflammatory syndrome*":ti,ab OR "inflammatory activ*":ti,ab OR "inflammatory disease*":ti,ab OR "systemic inflammat*":ti,ab OR "acute inflammat*":ti,ab OR "chronic inflammat*":ti,ab OR serositis:ti,ab OR "anti-inflammat*":ti,ab OR "anti-inflammatory effect*":ti,ab OR "anti-inflammatory activ*":ti,ab OR "antiinflammat*":ti,ab OR "antiinflammatory activ*":ti,ab OR "oxidative stress*":ti,ab OR "oxidative capacit*":ti,ab OR "oxidative damag*":ti,ab OR "oxidative balance*":ti,ab OR "oxidative stress-induced damag*":ti,ab OR "oxidative stress induced damag*":ti,ab OR "oxidation reduction stat*":ti,ab OR "oxidative DNA damag*":ti,ab OR damag*:ti,ab OR "oxidant stress*":ti,ab OR antioxida* OR anti-oxida*:ti,ab OR "anti oxida*":ti,ab OR "antioxidant effect*":ti,ab OR "anti-oxidant effect*":ti,ab OR "anti oxidant effect*":ti,ab OR "antioxidative effect*":ti,ab OR "anti-oxidative effect*":ti,ab OR "anti oxidative effect*":ti,ab OR "antioxidant capacit*":ti,ab OR "anti-oxidant capacit*":ti,ab OR "anti oxidant capacit*":ti,ab OR "antioxidative capacit*":ti,ab OR "anti-oxidative capacit*":ti,ab OR "anti oxidative capacit*":ti,ab OR "antioxidant activ*":ti,ab OR "anti-oxidant activ*":ti,ab OR "anti oxidant activ*":ti,ab OR "antioxidative activ*":ti,ab OR "anti-oxidative activ*":ti,ab OR "anti oxidative activ*":ti,ab OR "antioxidant response element*":ti,ab OR "anti oxidant response element*":ti,ab OR "anti-oxidant response element*":ti,ab OR "antioxidant assay*":ti,ab OR "anti-oxidant assay*":ti,ab OR "anti oxidant assay*":ti,ab OR "ferric	13509027

	reducing ability of plasma":ti,ab OR "antioxidant capacity assay*":ti,ab OR "antioxidant activity assay*":ti,ab OR "antioxidative assay*":ti,ab OR "oxygen radical absorbance capacity assay*":ti,ab OR "cell-based antioxidant protection assay*":ti,ab OR "thiobarbituric acid reactive substance assay*":ti,ab OR "lipid peroxidation assay*":ti,ab OR "free radical scavenging assay*":ti,ab)	
#3	(("randomised controlled trial":pt OR "controlled clinical trial":pt OR randomised:ab OR placebo:ab OR "clinical trials (topic)"/exp OR randomly:ab OR trial:ti) NOT (animals/exp NOT humans/exp))	840750
#4	#1 AND #2 AND #3	1258

**Scopus Search Strategy**		
**No.**	**Query**	**Results**
#1	TITLE-ABS(anthocyanin*) OR TITLE-ABS(anthocyanidin*) OR TITLE-ABS(leucoanthocyanidin*) OR TITLE-ABS("anthocyanin glycoside*") OR TITLE-ABS("anthocyanine glycoside*") OR TITLE-ABS("cassis anthocyanoside*") OR TITLE-ABS(cyanidin*) OR TITLE-ABS("cyanidin 3 glucoside*") OR TITLE-ABS("cyanidin 3-O-glucoside*") OR TITLE-ABS(delphinidin*) OR TITLE-ABS("delphinidin 3 glucoside*") OR TITLE-ABS("delphinidin 3-O-glucoside*") OR TITLE-ABS(pelargonidin* OR "pelargonidin 3 glucoside*") OR TITLE-ABS("pelargonidin 3-O-glucoside*") OR TITLE-ABS(peonidin*) OR TITLE-ABS("peonidin 3 glucoside*") OR TITLE-ABS("peonidin 3-O-glucoside*") OR TITLE-ABS(petunidin*) OR TITLE-ABS("petunidin 3 glucoside*") OR TITLE-ABS("petunidin 3-O-glucoside*") OR TITLE-ABS(malvidin*) OR TITLE-ABS("malvidin 3 glucoside*") OR TITLE-ABS("malvidin 3-O-glucoside*") OR TITLE-ABS("anthocyanin intake*") OR TITLE-ABS("anthocyanin consum*") OR TITLE-ABS(berry) OR TITLE-ABS(berries) OR TITLE-ABS(chokeberr*) OR TITLE-ABS(aronia) OR TITLE-ABS(melanocarpa) OR TITLE-ABS(aubergine*) OR TITLE-ABS(brinjal*) OR TITLE-ABS(eggplant*) OR TITLE-ABS("solanum melongena*") OR TITLE-ABS("Guinea squash") OR TITLE-ABS("Solanum insanum") OR TITLE-ABS("black currant*") OR TITLE-ABS("Ribes nigrum") OR TITLE-ABS(blueberr*) OR TITLE-ABS("Vaccinium corymbosum") OR TITLE-ABS("Vaccinium cyanococcus") OR TITLE-ABS("blood orange*") OR TITLE-ABS(cherry) OR TITLE-ABS(cherries) OR TITLE-ABS("Cerasus vulgaris") OR TITLE-ABS("Prunus cerasus") OR TITLE-ABS("Prunus avium") OR TITLE-ABS(grape*) OR TITLE-ABS(rhubarb) OR TITLE-ABS("rheum rhabarbarum") OR TITLE-ABS(strawberr*) OR TITLE-ABS("fragaria vesca") OR TITLE-ABS("Fragaria ananassa") OR TITLE-ABS(blackberr*) OR TITLE-ABS(raspberr*) OR TITLE-ABS("rubus glaucus") OR TITLE-ABS("Rubus fruticosus") OR TITLE-ABS(plum*) OR TITLE-ABS("red cabbage*" OR "purple cabbage*") OR TITLE-ABS("Brassica oleracea var capitata f rubra") OR TITLE-ABS("red wine") OR TITLE-ABS(cranberr*) OR TITLE-ABS("vaccinium macrocarpon*") OR TITLE-ABS(elderberr*) OR TITLE-ABS("sambucus Canadensis") OR TITLE-ABS(bilberr*) OR TITLE-ABS("vaccinium myrtillus") OR TITLE-ABS(whortleberr*)	240575
#2	(TITLE-ABS("cardiovascular disease*") OR TITLE-ABS("atherosclerotic cardiovascular disease*") OR TITLE-ABS("cardiovascular risk*") OR TITLE-ABS("cardio-vascular disease*") OR TITLE-ABS(angiocardiopath*) OR TITLE-ABS("angiocardiovascular disease*") OR TITLE-ABS("cardiovascular complication*") OR TITLE-ABS("cardiovascular disorder*") OR TITLE-ABS("cardiovascular disturbance*") OR TITLE-ABS("cardiovascular lesion*") OR TITLE-ABS("cardiovascular syndrome*") OR TITLE-ABS("cardiovascular vegetative disorder*") OR TITLE-ABS("cardiovascular complication*") OR TITLE-ABS("major adverse cardiovascular event*") OR TITLE-ABS("glucose metabolism disorder*") OR TITLE-ABS("glucose metabolic disorder*") OR TITLE-ABS("biotin responsive multiple carboxylase deficienc*") OR TITLE-ABS("combined carboxylase deficienc*") OR TITLE-ABS("glucose metabolism disorder*") OR TITLE-ABS("holocarboxylase deficienc*") OR TITLE-ABS("holocarboxylase synthetase deficienc*") OR TITLE-ABS("mckusick 25327") OR TITLE-ABS("multiple carboxylase deficienc*") OR TITLE-ABS("multiple carboxylase deficienc*") OR TITLE-ABS("lipid metabolism disorder*") OR TITLE-ABS("lipid metabolic disorder*") OR TITLE-ABS("nutritional disorder*") OR TITLE-ABS("metabolic syndrome*") OR TITLE-ABS("metabolic syndrome X") OR TITLE-ABS("insulin resistance syndrome X") OR TITLE-ABS("metabolic X syndrome") OR TITLE-ABS("dysmetabolic syndrome X") OR TITLE-ABS("reaven syndrome X") OR TITLE-ABS("metabolic cardiovascular syndrome*") OR TITLE-ABS("body function disorder*") OR TITLE-ABS("pathologic process*") OR TITLE-ABS("pathological condition*") OR TITLE-ABS("symptoms and general pathology") OR TITLE-ABS(inflammat*) OR TITLE-ABS("inflammation reaction*") OR TITLE-ABS("inflammatory reaction*") OR TITLE-ABS("inflammation response*") OR TITLE-ABS("inflammatory response*") OR TITLE-ABS("inflammatory condition*") OR TITLE-ABS("inflammatory lesion*") OR TITLE-ABS("inflammatory process*") OR TITLE-ABS("inflammatory syndrome*") OR TITLE-ABS("inflammatory activ*") OR TITLE-ABS("inflammatory disease*") OR TITLE-ABS("systemic inflammat*") OR TITLE-ABS("acute inflammat*") OR TITLE-ABS("chronic inflammat*") OR TITLE-ABS(serositis) OR TITLE-ABS("anti-inflammat*") OR TITLE-ABS("anti-inflammatory effect*") OR TITLE-ABS("anti-inflammatory activ*") OR TITLE-ABS("antiinflammat*") OR TITLE-ABS("antiinflammatory activ*") OR	2737679

	TITLE-ABS("oxidative stress*") OR TITLE-ABS("oxidative capacit*") OR TITLE-ABS("oxidative damag*") OR TITLE-ABS("oxidative balance*") OR TITLE-ABS("oxidative stress-induced damag*") OR TITLE-ABS("oxidative stress induced damag*") OR TITLE-ABS("oxidation reduction stat*") OR TITLE-ABS("oxidative DNA damag*") OR TITLE-ABS(damag*) OR TITLE-ABS("oxidant stress*") OR TITLE-ABS(antioxida* OR anti-oxida*) OR TITLE-ABS("anti oxida*") OR TITLE-ABS("antioxidant effect*") OR TITLE-ABS("anti-oxidant effect*") OR TITLE-ABS("anti oxidant effect*") OR TITLE-ABS("antioxidative effect*") OR TITLE-ABS("anti-oxidative effect*") OR TITLE-ABS("anti oxidative effect*") OR TITLE-ABS("antioxidant capacit*") OR TITLE-ABS("anti-oxidant capacit*") OR TITLE-ABS("anti oxidant capacit*") OR TITLE-ABS("antioxidative capacit*") OR TITLE-ABS("anti-oxidative capacit*") OR TITLE-ABS("anti oxidative capacit*") OR TITLE-ABS("antioxidant activ*") OR TITLE-ABS("anti-oxidant activ*") OR TITLE-ABS("anti oxidant activ*") OR TITLE-ABS("antioxidative activ*") OR TITLE-ABS("anti-oxidative activ*") OR TITLE-ABS("anti oxidative activ*") OR TITLE-ABS("antioxidant response element*") OR TITLE-ABS("anti oxidant response element*") OR TITLE-ABS("anti-oxidant response element*") OR TITLE-ABS("antioxidant assay*") OR TITLE-ABS("anti-oxidant assay*") OR TITLE-ABS("anti oxidant assay*") OR TITLE-ABS("ferric reducing ability of plasma") OR TITLE-ABS("antioxidant capacity assay*") OR TITLE-ABS("antioxidant activity assay*") OR TITLE-ABS("antioxidative assay*") OR TITLE-ABS("oxygen radical absorbance capacity assay*") OR TITLE-ABS("cell-based antioxidant protection assay*") OR TITLE-ABS("thiobarbituric acid reactive substance assay*") OR TITLE-ABS("lipid peroxidation assay*") OR TITLE-ABS("free radical scavenging assay*"))	
#3	(INDEXTERMS ("clinical trials" OR "clinical trials as a topic" OR "randomized controlled trial" OR "Randomized Controlled Trials as Topic" OR "controlled clinical trial" OR "Controlled Clinical Trials" OR "random allocation" OR "Double-Blind Method" OR "Single-Blind Method" OR "Cross-Over Studies" OR "Placebos" OR "multicenter study" OR "double blind procedure" OR "single blind procedure" OR "crossover procedure" OR "clinical trial" OR "controlled study" OR "randomization" OR "placebo")) OR (TITLE-ABS-KEY (("clinical trials" OR "clinical trials as a topic" OR "randomized controlled trial" OR "Randomized Controlled Trials as Topic" OR "controlled clinical trial" OR "Controlled Clinical Trials as Topic" OR "random allocation" OR "randomly allocated" OR "allocated randomly" OR "Double-Blind Method" OR "Single-Blind Method" OR "Cross-Over Studies" OR "Placebos" OR "cross-over trial" OR "single blind" OR "double blind" OR "factorial design" OR "factorial trial"))) OR (TITLE-ABS (clinical trial* OR trial* OR rct* OR random* OR blind*))	7249083
#4	#1 AND #2 AND #3	6114

**CINAHL Search Strategy**		
**No.**	**Query**	**Results**
#1	((MH "Berries") OR "berries" OR (MH "Blackberry") OR (MH "Elderberry") OR (MH "Blueberry") OR (MH "Bilberry") OR (MH "Wild Cherry") OR (MH "Sour Cherry") OR (MH "Cherries") OR (MH "Rhubarb") OR (MH "Strawberry") OR (MH "Raspberry") OR (MH "Cranberry") OR (MH "Cranberry Juice")) OR (TI anthocyanin* OR TI anthocyanidin* OR TI leucoanthocyanidin* OR TI "anthocyanin glycoside*" OR TI "anthocyanine glycoside*" OR TI "cassis anthocyanoside*" OR TI cyanidin* OR TI "cyanidin 3 glucoside*" OR TI "cyanidin 3-O-glucoside*" OR TI delphinidin* OR TI "delphinidin 3 glucoside*" OR TI "delphinidin 3-O-glucoside*" OR TI pelargonidin* OR "pelargonidin 3 glucoside*" OR TI "pelargonidin 3-O-glucoside*" OR TI peonidin* OR TI "peonidin 3 glucoside*" OR TI "peonidin 3-O-glucoside*" OR TI petunidin* OR TI "petunidin 3 glucoside*" OR TI "petunidin 3-O-glucoside*" OR TI malvidin* OR TI "malvidin 3 glucoside*" OR TI "malvidin 3-O-glucoside*" OR TI "anthocyanin intake*" OR TI "anthocyanin consum*" OR TI berry OR TI berries OR TI chokeberr* OR TI aronia OR TI melanocarpa OR TI aubergine* OR TI brinjal* OR TI eggplant* OR TI "solanum melongena*" OR TI "Guinea squash" OR TI "Solanum insanum" OR TI "black currant*" OR TI "Ribes nigrum" OR TI blueberr* OR TI "Vaccinium corymbosum" OR TI "Vaccinium cyanococcus" OR TI "blood orange*" OR TI cherry OR TI cherries OR TI "Cerasus vulgaris" OR TI "Prunus cerasus" OR TI "Prunus avium" OR TI grape* OR TI rhubarb OR TI "rheum rhabarbarum" OR TI strawberr* OR TI "fragaria vesca" OR TI "Fragaria ananassa" OR TI blackberr* OR TI raspberr* OR TI "rubus glaucus" OR TI "Rubus fruticosus" OR TI plum* OR TI "red cabbage*" OR "purple cabbage*" OR TI "Brassica oleracea var capitata f rubra" OR TI "red wine" OR TI cranberr* OR TI "vaccinium macrocarpon*" OR TI elderberr* OR TI "sambucus Canadensis" OR TI bilberr* OR TI "vaccinium myrtillus" OR TI whortleberr* OR AB anthocyanin* OR AB anthocyanidin* OR AB leucoanthocyanidin* OR AB "anthocyanin glycoside*" OR AB "anthocyanine glycoside*" OR AB "cassis anthocyanoside*" OR AB cyanidin* OR AB "cyanidin 3 glucoside*" OR AB "cyanidin 3-O-glucoside*" OR AB delphinidin* OR AB "delphinidin 3 glucoside*" OR AB "delphinidin 3-O-glucoside*" OR AB pelargonidin* OR "pelargonidin 3 glucoside*" OR AB "pelargonidin 3-O-glucoside*" OR AB peonidin* OR AB "peonidin 3 glucoside*" OR AB "peonidin 3-O-glucoside*" OR AB petunidin* OR AB "petunidin 3 glucoside*" OR AB "petunidin 3-O-glucoside*" OR AB malvidin* OR AB "malvidin 3 glucoside*" OR AB "malvidin 3-O-glucoside*" OR AB "anthocyanin intake*" OR AB "anthocyanin consum*" OR AB berry OR	13949

	AB berries OR AB chokeberr* OR AB aronia OR AB melanocarpa OR AB aubergine* OR AB brinjal* OR AB eggplant* OR AB "solanum melongena*" OR AB "Guinea squash" OR AB "Solanum insanum" OR AB "black currant*" OR AB "Ribes nigrum" OR AB blueberr* OR AB "Vaccinium corymbosum" OR AB "Vaccinium cyanococcus" OR AB "blood orange*" OR AB cherry OR AB cherries OR AB "Cerasus vulgaris" OR AB "Prunus cerasus" OR AB "Prunus avium" OR AB grape* OR AB rhubarb OR AB "rheum rhabarbarum" OR AB strawberr* OR AB "fragaria vesca" OR AB "Fragaria ananassa" OR AB blackberr* OR AB raspberr* OR AB "rubus glaucus" OR AB "Rubus fruticosus" OR AB plum* OR AB "red cabbage*" OR "purple cabbage*" OR AB "Brassica oleracea var capitata f rubra" OR AB "red wine" OR AB cranberr* OR AB "vaccinium macrocarpon*" OR AB elderberr* OR AB "sambucus Canadensis" OR AB bilberr* OR AB "vaccinium myrtillus" OR AB whortleberr*)	
S5	((MH "Cardiovascular Diseases+") OR (MH "Metabolic Diseases+") OR (MH "Nutrition Disorders+") OR (MH "Inflammation+") OR (MH "Oxidative Stress") OR (MH "Antioxidants+")) OR (TI "cardiovascular disease*" OR TI "atherosclerotic cardiovascular disease*" OR TI "cardiovascular risk*" OR TI "cardio-vascular disease*" OR TI angiocardiopath* OR TI "angiocardiovascular disease*" OR TI "cardiovascular complication*" OR TI "cardiovascular disorder*" OR TI "cardiovascular disturbance*" OR TI "cardiovascular lesion*" OR TI "cardiovascular syndrome*" OR TI "cardiovascular vegetative disorder*" OR TI "cardiovascular complication*" OR TI "major adverse cardiovascular event*" OR TI "glucose metabolism disorder*" OR TI "glucose metabolic disorder*" OR TI "biotin responsive multiple carboxylase deficienc*" OR TI "combined carboxylase deficienc*" OR TI "glucose metabolism disorder*" OR TI "holocarboxylase deficienc*" OR TI "holocarboxylase synthetase deficienc*" OR TI "mckusick 25327" OR TI "multiple carboxylase deficienc*" OR TI "multiple carboxylase deficienc*" OR TI "lipid metabolism disorder*" OR TI "lipid metabolic disorder*" OR TI "nutritional disorder*" OR TI "metabolic syndrome*" OR TI "metabolic syndrome X" OR TI "insulin resistance syndrome X" OR TI "metabolic X syndrome" OR TI "dysmetabolic syndrome X" OR TI "reaven syndrome X" OR TI "metabolic cardiovascular syndrome*" OR TI "body function disorder*" OR TI "pathologic process*" OR TI "pathological condition*" OR TI "symptoms and general pathology" OR TI inflammat* OR TI "inflammation reaction*" OR TI "inflammatory reaction*" OR TI "inflammation response*" OR TI "inflammatory response*" OR TI "inflammatory condition*" OR TI "inflammatory lesion*" OR TI "inflammatory process*" OR TI "inflammatory syndrome*" OR TI "inflammatory activ*" OR TI "inflammatory disease*" OR TI "systemic inflammat*" OR TI "acute inflammat*" OR TI "chronic inflammat*" OR TI serositis OR TI "anti-inflammat*" OR TI "anti-inflammatory effect*" OR TI "anti-inflammatory activ*" OR TI "antiinflammat*" OR TI "antiinflammatory activ*" OR TI "oxidative stress*" OR TI "oxidative capacit*" OR TI "oxidative damag*" OR TI "oxidative balance*" OR TI "oxidative stress-induced damag*" OR TI "oxidative stress induced damag*" OR TI "oxidation reduction stat*" OR TI "oxidative DNA damag*" OR TI damag* OR TI "oxidant stress*" OR TI antioxida* OR anti-oxida* OR TI "anti oxida*" OR TI "antioxidant effect*" OR TI "anti-oxidant effect*" OR TI "anti oxidant effect*" OR TI "antioxidative effect*" OR TI "anti-oxidative effect*" OR TI "anti oxidative effect*" OR TI "antioxidant capacit*" OR TI "anti-oxidant capacit*" OR TI "anti oxidant capacit*" OR TI "antioxidative capacit*" OR TI "anti-oxidative capacit*" OR TI "anti oxidative capacit*" OR TI "antioxidant activ*" OR TI "anti-oxidant activ*" OR TI "anti oxidant activ*" OR TI "antioxidative activ*" OR TI "anti-oxidative activ*" OR TI "anti oxidative activ*" OR TI "antioxidant response element*" OR TI "anti oxidant response element*" OR TI "anti-oxidant response element*" OR TI "antioxidant assay*" OR TI "anti-oxidant assay*" OR TI "anti oxidant assay*" OR TI "ferric reducing ability of plasma" OR TI "antioxidant capacity assay*" OR TI "antioxidant activity assay*" OR TI "antioxidative assay*" OR TI "oxygen radical absorbance capacity assay*" OR TI "cell-based antioxidant protection assay*" OR TI "thiobarbituric acid reactive substance assay*" OR TI "lipid peroxidation assay*" OR TI "free radical scavenging assay*" OR AB "cardiovascular disease*" OR AB "atherosclerotic cardiovascular disease*" OR AB "cardiovascular risk*" OR AB "cardio-vascular disease*" OR AB angiocardiopath* OR AB "angiocardiovascular disease*" OR AB "cardiovascular complication*" OR AB "cardiovascular disorder*" OR AB "cardiovascular disturbance*" OR AB "cardiovascular lesion*" OR AB "cardiovascular syndrome*" OR AB "cardiovascular vegetative disorder*" OR AB "cardiovascular complication*" OR AB "major adverse cardiovascular event*" OR AB "glucose metabolism disorder*" OR AB "glucose metabolic disorder*" OR AB "biotin responsive multiple carboxylase deficienc*" OR AB "combined carboxylase deficienc*" OR AB "glucose metabolism disorder*" OR AB "holocarboxylase deficienc*" OR AB "holocarboxylase synthetase deficienc*" OR AB "mckusick 25327" OR AB "multiple carboxylase deficienc*" OR AB "multiple carboxylase deficienc*" OR AB "lipid metabolism disorder*" OR AB "lipid metabolic disorder*" OR AB "nutritional disorder*" OR AB "metabolic syndrome*" OR AB "metabolic syndrome X" OR AB "insulin resistance syndrome X" OR AB "metabolic X syndrome" OR AB "dysmetabolic syndrome X" OR AB "reaven syndrome X" OR AB "metabolic cardiovascular syndrome*" OR AB "body function disorder*" OR AB "pathologic process*" OR AB "pathological condition*" OR AB "symptoms and general pathology" OR AB inflammat* OR AB "inflammation reaction*" OR AB "inflammatory reaction*" OR AB "inflammation response*" OR AB "inflammatory response*" OR AB "inflammatory condition*" OR AB "inflammatory lesion*" OR AB "inflammatory process*" OR AB "inflammatory syndrome*" OR AB "inflammatory activ*" OR AB "inflammatory disease*" OR AB "systemic inflammat*" OR AB "acute inflammat*" OR AB "chronic inflammat*" OR AB serositis OR AB "anti-inflammat*" OR AB "anti-inflammatory effect*" OR AB "anti-inflammatory activ*" OR AB "antiinflammat*" OR AB "antiinflammatory activ*" OR AB "oxidative stress*" OR AB "oxidative capacit*" OR AB "oxidative damag*" OR AB "oxidative balance*" OR AB "oxidative stress-induced damag*" OR AB "oxidative stress induced damag*" OR AB "oxidation reduction stat*" OR AB "oxidative DNA damag*" OR AB damag* OR AB "oxidant stress*" OR AB antioxida* OR anti-oxida* OR AB "anti oxida*" OR AB "antioxidant effect*" OR AB "anti-oxidant	971873

	effect*" OR AB "anti oxidant effect*" OR AB "antioxidative effect*" OR AB "anti-oxidative effect*" OR AB "anti oxidative effect*" OR AB "antioxidant capacit*" OR AB "anti-oxidant capacit*" OR AB "anti oxidant capacit*" OR AB "antioxidative capacit*" OR AB "anti-oxidative capacit*" OR AB "anti oxidative capacit*" OR AB "antioxidant activ*" OR AB "anti-oxidant activ*" OR AB "anti oxidant activ*" OR AB "antioxidative activ*" OR AB "anti-oxidative activ*" OR AB "anti oxidative activ*" OR AB "antioxidant response element*" OR AB "anti oxidant response element*" OR AB "anti-oxidant response element*" OR AB "antioxidant assay*" OR AB "anti-oxidant assay*" OR AB "anti oxidant assay*" OR AB "ferric reducing ability of plasma" OR AB "antioxidant capacity assay*" OR AB "antioxidant activity assay*" OR AB "antioxidative assay*" OR AB "oxygen radical absorbance capacity assay*" OR AB "cell-based antioxidant protection assay*" OR AB "thiobarbituric acid reactive substance assay*" OR AB "lipid peroxidation assay*" OR AB "free radical scavenging assay*")	
S7	S3 AND S6	4376
S8	Limiters - Publication Type: Randomised Controlled Trials	188

**Cochrane Search Strategy**		
**No.**	**Query**	**Results**
#1	MeSH descriptor: [Anthocyanins] explode all trees	116
#2	MeSH descriptor: [Solanum melongena] this term only	1
#3	MeSH descriptor: [Fragaria] this term only	35
#4	MeSH descriptor: [Rubus] this term only	12
#5	MeSH descriptor: [Vaccinium macrocarpon] this term only	94
#6	MeSH descriptor: [Sambucus] this term only	9
#7	MeSH descriptor: [Vaccinium myrtillus] this term only	26
#8	(anthocyanin*:ti,ab OR anthocyanidin*:ti,ab OR leucoanthocyanidin*:ti,ab OR "anthocyanin glycoside*":ti,ab OR "anthocyanine glycoside*":ti,ab OR "cassis anthocyanoside*":ti,ab OR cyanidin*:ti,ab OR "cyanidin 3 glucoside*":ti,ab OR "cyanidin 3-O-glucoside*":ti,ab OR delphinidin*:ti,ab OR "delphinidin 3 glucoside*":ti,ab OR "delphinidin 3-O-glucoside*":ti,ab OR pelargonidin* OR "pelargonidin 3 glucoside*":ti,ab OR "pelargonidin 3-O-glucoside*":ti,ab OR peonidin*:ti,ab OR "peonidin 3 glucoside*":ti,ab OR "peonidin 3-O-glucoside*":ti,ab OR petunidin*:ti,ab OR "petunidin 3 glucoside*":ti,ab OR "petunidin 3-O-glucoside*":ti,ab OR malvidin*:ti,ab OR "malvidin 3 glucoside*":ti,ab OR "malvidin 3-O-glucoside*":ti,ab OR "anthocyanin intake*":ti,ab OR "anthocyanin consum*":ti,ab OR berry:ti,ab OR berries:ti,ab OR chokeberr*:ti,ab OR aronia:ti,ab OR melanocarpa:ti,ab OR aubergine*:ti,ab OR brinjal*:ti,ab OR eggplant*:ti,ab OR "solanum melongena*":ti,ab OR "Guinea squash":ti,ab OR "Solanum insanum":ti,ab OR "black currant*":ti,ab OR "Ribes nigrum":ti,ab OR blueberr*:ti,ab OR "Vaccinium corymbosum":ti,ab OR "Vaccinium cyanococcus":ti,ab OR "blood orange*":ti,ab OR cherry:ti,ab OR cherries:ti,ab OR "Cerasus vulgaris":ti,ab OR "Prunus cerasus":ti,ab OR "Prunus avium":ti,ab OR grape*:ti,ab OR rhubarb:ti,ab OR "rheum rhabarbarum":ti,ab OR strawberr*:ti,ab OR "fragaria vesca":ti,ab OR "Fragaria ananassa":ti,ab OR blackberr*:ti,ab OR raspberr*:ti,ab OR "rubus glaucus":ti,ab OR "Rubus fruticosus":ti,ab OR plum*:ti,ab OR "red cabbage*" OR "purple cabbage*":ti,ab OR "Brassica oleracea var capitata f rubra":ti,ab OR "red wine":ti,ab OR cranberr*:ti,ab OR "vaccinium macrocarpon*":ti,ab OR elderberr*:ti,ab OR "sambucus Canadensis":ti,ab OR bilberr*:ti,ab OR "vaccinium myrtillus":ti,ab OR whortleberr*:ti,ab)	3536
#9	#1 OR #2 OR #3 OR #4 OR #5 OR #6 OR #7 OR #8	3554

#10	MeSH descriptor: [Cardiovascular Diseases] explode all trees	98575
#11	MeSH descriptor: [Glucose Metabolism Disorders] explode all trees	30070
#12	MeSH descriptor: [Lipid Metabolism Disorders] explode all trees	7247
#13	MeSH descriptor: [Metabolic syndrome] explode all trees	1631
#14	MeSH decriptor [Inflammation] explode all trees	9884
#15	MeSH decriptor: [Oxidative stress] explode all trees	2914
#16	MeSH descriptor: [Antioxidants] explode all trees	4667
#17	"cardiovascular disease*":ti,ab OR "atherosclerotic cardiovascular disease*":ti,ab OR "cardiovascular risk*":ti,ab OR "cardio-vascular disease*":ti,ab OR angiocardiopath*:ti,ab OR "angiocardiovascular disease*":ti,ab OR "cardiovascular complication*":ti,ab OR "cardiovascular disorder*":ti,ab OR "cardiovascular disturbance*":ti,ab OR "cardiovascular lesion*":ti,ab OR "cardiovascular syndrome*":ti,ab OR "cardiovascular vegetative disorder*":ti,ab OR "cardiovascular complication*":ti,ab OR "major adverse cardiovascular event*":ti,ab OR "glucose metabolism disorder*":ti,ab OR "glucose metabolic disorder*":ti,ab OR "biotin responsive multiple carboxylase deficienc*":ti,ab OR "combined carboxylase deficienc*":ti,ab OR "glucose metabolism disorder*":ti,ab OR "holocarboxylase deficienc*":ti,ab OR "holocarboxylase synthetase deficienc*":ti,ab OR "mckusick 25327":ti,ab OR "multiple carboxylase deficienc*":ti,ab OR "multiple carboxylase deficienc*":ti,ab OR "lipid metabolism disorder*":ti,ab OR "lipid metabolic disorder*":ti,ab OR "nutritional disorder*":ti,ab OR "metabolic syndrome*":ti,ab OR "metabolic syndrome X":ti,ab OR "insulin resistance syndrome X":ti,ab OR "metabolic X syndrome":ti,ab OR "dysmetabolic syndrome X":ti,ab OR "reaven syndrome X":ti,ab OR "metabolic cardiovascular syndrome*":ti,ab OR "body function disorder*":ti,ab OR "pathologic process*":ti,ab OR "pathological condition*":ti,ab OR "symptoms and general pathology":ti,ab OR inflammat*:ti,ab OR "inflammation reaction*":ti,ab OR "inflammatory reaction*":ti,ab OR "inflammation response*":ti,ab OR "inflammatory response*":ti,ab OR "inflammatory condition*":ti,ab OR "inflammatory lesion*":ti,ab OR "inflammatory process*":ti,ab OR "inflammatory syndrome*":ti,ab OR "inflammatory activ*":ti,ab OR "inflammatory disease*":ti,ab OR "systemic inflammat*":ti,ab OR "acute inflammat*":ti,ab OR "chronic inflammat*":ti,ab OR serositis:ti,ab OR "anti-inflammat*":ti,ab OR "anti-inflammatory effect*":ti,ab OR "anti-inflammatory activ*":ti,ab OR "antiinflammat*":ti,ab OR "antiinflammatory activ*":ti,ab OR "oxidative stress*":ti,ab OR "oxidative capacit*":ti,ab OR "oxidative damag*":ti,ab OR "oxidative balance*":ti,ab OR "oxidative stress-induced damag*":ti,ab OR "oxidative stress induced damag*":ti,ab OR "oxidation reduction stat*":ti,ab OR "oxidative DNA damag*":ti,ab OR damag*:ti,ab OR "oxidant stress*":ti,ab OR antioxida* OR anti-oxida*:ti,ab OR "anti oxida*":ti,ab OR "antioxidant effect*":ti,ab OR "anti-oxidant effect*":ti,ab OR "anti oxidant effect*":ti,ab OR "antioxidative effect*":ti,ab OR "anti-oxidative effect*":ti,ab OR "anti oxidative effect*":ti,ab OR "antioxidant capacit*":ti,ab OR "anti-oxidant capacit*":ti,ab OR "anti oxidant capacit*":ti,ab OR "antioxidative capacit*":ti,ab OR "anti-oxidative capacit*":ti,ab OR "anti oxidative capacit*":ti,ab OR "antioxidant activ*":ti,ab OR "anti-oxidant activ*":ti,ab OR "anti oxidant activ*":ti,ab OR "antioxidative activ*":ti,ab OR "anti-oxidative activ*":ti,ab OR "anti oxidative activ*":ti,ab OR "antioxidant response element*":ti,ab OR "anti oxidant response element*":ti,ab OR "anti-oxidant response element*":ti,ab OR "antioxidant assay*":ti,ab OR "anti-oxidant assay*":ti,ab OR "anti oxidant assay*":ti,ab OR "ferric reducing ability of plasma":ti,ab OR "antioxidant capacity assay*":ti,ab OR "antioxidant activity assay*":ti,ab OR "antioxidative assay*":ti,ab OR "oxygen radical absorbance capacity assay*":ti,ab OR "cell-based antioxidant protection assay*":ti,ab OR "thiobarbituric acid reactive substance assay*":ti,ab OR "lipid peroxidation assay*":ti,ab OR "free radical scavenging assay*":ti,ab	115333
#18	#10 OR #11 OR #12 OR #13 OR #14 OR #15 OR #16 OR #17	228556
#19	#9 and #18	1313
